# Identification of Yeast Genes Involved in K^+^ Homeostasis: Loss of Membrane Traffic Genes Affects K^+^ Uptake

**DOI:** 10.1534/g3.111.000166

**Published:** 2011-06-01

**Authors:** Gillian L. Fell, Amanda M. Munson, Merriah A. Croston, Anne G. Rosenwald

**Affiliations:** Department of Biology, Georgetown University, Washington, DC 20057

**Keywords:** *VPS* genes, *TRK1*

## Abstract

Using the homozygous diploid *Saccharomyces* deletion collection, we searched for strains with defects in K^+^ homeostasis. We identified 156 (of 4653 total) strains unable to grow in the presence of hygromycin B, a phenotype previously shown to be indicative of ion defects. The most abundant group was that with deletions of genes known to encode membrane traffic regulators. Nearly 80% of these membrane traffic defective strains showed defects in uptake of the K^+^ homolog, ^86^Rb^+^. Since Trk1, a plasma membrane protein localized to lipid microdomains, is the major K^+^ influx transporter, we examined the subcellular localization and Triton-X 100 insolubility of Trk1 in 29 of the traffic mutants. However, few of these showed defects in the steady state levels of Trk1, the localization of Trk1 to the plasma membrane, or the localization of Trk1 to lipid microdomains, and most defects were mild compared to wild-type. Three inositol kinase mutants were also identified, and in contrast, loss of these genes negatively affected Trk1 protein levels. In summary, this work reveals a nexus between K^+^ homeostasis and membrane traffic, which does not involve traffic of the major influx transporter, Trk1.

K^+^ is the major cation in cells, present at submolar concentrations (0.05–0.2 M) in the cytoplasm, concentrated ∼1000-fold relative to the external environment (Rodriguez-Navarro, 2000, Perkins and Gadd 1993). High intracellular levels of K^+^ are required for replication and translation (Lubin, 1964, Lubin and Ennis, 1964, Lubin 1967). Further, K^+^ is required for uptake of nutrients, disposal of metabolic by-products, and cell–cell communication in multicellular organisms, particularly in neural tissue. In addition, asymmetric K^+^ fluxes very early in vertebrate development are required for left-right asymmetry (Levin *et al.*, 2002, Raya *et al.*, 2004), dependent upon Kir4.1 and the H^+^/K^+^-ATPase (Aw *et al.*, 2008), while later in development, formation of the neural crest depends upon the activity of cation/H^+^-antiporters (Manohar *et al.*, 2010). In yeast at least, K^+^ is also required for cell cycle progression (Masuda *et al.*, 2000), but it is not currently known whether this is a feature of cell cycle control in higher eukaryotes.

In yeast, K^+^ import is accomplished primarily by the K^+^ influx transporter, Trk1 (Gaber, 1992, Ko *et al.*, 1990, Ko and Gaber, 1991, Ramos *et al.*, 1994). A paralog of Trk1, named Trk2, has been referred to as a low- or medium-affinity transporter (Ko *et al.*, 1990), which is apparently only expressed under specific conditions, including low external K^+^ or low external pH (Michel *et al.*, 2006). The protein Qdr2 may also play a minor role in Trk-independent K^+^ uptake (Vargas *et al.*, 2007). *trk1Δtrk2Δ* mutants are sensitive to toxic cations including hygromycin B and show deficient K^+^ uptake (Madrid *et al.*, 1998, Navarrete *et al.*, 2010). *sat4/hal4Δhal5Δ* double mutants also exhibit cation sensitivity and deficient K^+^ uptake (Pérez-Valle *et al.*, 2007). Overexpression of *SAT4/HAL4* or *HAL5*, which encode serine/threonine kinases, increases tolerance to toxic cations; however, overexpression of these genes in a *trk1Δtrk2Δ* background is without effect, suggesting that Hal4 and Hal5 act to regulate the activity or stability of Trk1 and Trk2 (Mulet *et al.*, 1999). Recent work also implicates calcineurin as a factor in the regulation of Trk1 via the Hal proteins (Casado *et al.*, 2010).

Other regulators of Trk-dependent K^+^ import include the serine/threonine phosphatases Ppz1 and Ppz2. The Ppz proteins have diverse functions in ion homeostasis, cell wall maintenance, and regulation of cell growth and division (Posas *et al.*, 1995, Clotet *et al.*, 1999). *PPZ1* overexpression decreases uptake of the K^+^ homolog, ^86^Rb^+^, while *ppz1Δppz2Δ* mutants are resistant to toxic cations (Yenush *et al.*, 2002). Hal3 directly interacts with the Ppz proteins, decreasing their phosphatase activity, and cells lacking functional Hal3 demonstrate decreased ^86^Rb^+^ uptake compared to wild-type cells (Yenush *et al.*, 2002, De Nadal *et al.*, 1999, Yenush *et al.*, 2005). Vhs3, homologous to Hal3, also negatively regulates Ppz1 activity (Ruiz *et al.*, 2004a).

In addition to the kinases and phosphatases mentioned above, we previously found that loss of the guanine-nucleotide binding protein gene, *ARL1*, causes increased sensitivity to toxic cations including hygromcyin B, correlating with decreased K^+^ influx, which is suppressed by additional K^+^ in the medium (Munson *et al.*, 2004a). Although Arl1 in both yeast and mammals has documented roles in regulation of membrane traffic (Rosenwald *et al.*, 2002, Love *et al.*, 2004, Lu *et al.*, 2001, Lowe *et al.*, 1996, Munro, 2005, Panic *et al.*, 2003, Liu *et al.*, 2005, Behnia *et al.*, 2004), Arl1 does not control traffic of Trk1: loss of *ARL1* has no effect on steady levels or localization of Trk1 to the plasma membrane (Munson *et al.*, 2004a). In addition, loss of *ARL1* has no effect on localization of Trk1 to lipid microdomains (this work). To identify other genes which potentially play a role K^+^ homeostasis, we screened the homozygous diploid deletion collection of viable yeast mutants (Winzeler *et al.*, 1999) for strains exhibiting a hygromycin B-sensitive phenotype. We identified a number of such mutants and found that many of them had deletions of genes previously shown to encode regulators of membrane traffic.

The connection between K^+^ and membrane traffic was first identified more than 20 years ago, when it was shown that K^+^ was important in mammalian cells for clathrin-dependent endocytosis (Heuser and Anderson, 1989, Larkin *et al.*, 1986, Larkin *et al.*, 1985, Larkin *et al.*, 1983). It has been suggested that K^+^ is required for the activity of the clathrin uncoating ATPase, Hsc70 (O'Brien and Mckay, 1995, Wilbanks and Mckay 1995). Newer data show that depletion of intracellular K^+^ also affects clathrin-independent endocytosis (mediated primarily by caveolae) (Vercauteren *et al.*, 2010) and that effects of K^+^-depletion on endocytic functions are cell-type specific (Vercauteren *et al.*, 2010), suggesting there is at least one other mechanism responsible in addition to possible inhibition of the uncoating ATPase. Work in mammalian cells has also shown that aminoglycoside antibiotics like hygromycin B bind to coatomer subunits (Hudson and Draper 1997). Membrane traffic in yeast is controlled by regulation of cytosolic *vs.* lumenal pH and K^+^ specifically by the activity of Nhx1/Vps44, a Na^+^-K^+^/H^+^ exchanger localized to late endosomal membranes (Ali *et al.*, 2004, Brett *et al.*, 2005). Together, these data demonstrate a role for K^+^ in ensuring effective membrane trafficking. It would therefore be reasonable for gene products involved in membrane traffic to have a role in modulating K^+^ for the purpose of optimizing membrane traffic. Indeed, one previous study suggested a role for the SNARE protein SYP121 in K^+^ channel gating in *Arabidopsis* (Honsbein *et al.*, 2009). In general, however, the molecular details that underlie connections between K^+^ homeostasis and membrane traffic have not been fully elucidated.

In this paper, therefore, by identification of numerous membrane traffic regulators as mediators of K^+^ influx, we have made a step toward delineating these connections. While others have also discovered that membrane traffic mutants are sensitive to hygromycin B (recent examples include [Banuelos *et al.* (2010), Dudley *et al.* (2005), and Conboy and Cyert (2000)], the novel aspects of this work are the findings that many of these mutants show deficient K^+^ uptake and that some phenotypes can be suppressed by the addition of excess K^+^ in the medium. However, for the majority of mutants examined, although defective for K^+^ uptake, wild-type levels of Trk1, correct localization of Trk1 to the plasma membrane fraction, and correct localization to lipid microdomains were observed.

Thus, our new results demonstrate that a number of different membrane traffic genes in addition to Arl1 also regulate K^+^ uptake. However, our results are consistent with a model in which defective regulation of Trk1 activity occurs rather than defective localization.

## Materials and Methods

### Strains and plasmids

The 4653 homozygous diploid mutant strains used in this study were obtained from OpenBiosystems (formerly Research Genetics, Huntsville, AL). Each *S. cerevisiae* strain in the collection lacks both copies of a single nonessential gene (Winzeler *et al.*, 1999). The parental strain is BY4743 (*MATα/MATa his3Δ1/his3Δ1 leu2Δ0/leu2Δ0 +/ met15Δ0 +/lys2Δ0 ura3Δ0/ura3Δ0*). The web site http://www-sequence.stanford.edu/group/yeast_deletion_project/deletions3.html provides further information about the collection. Briefly, a PCR-based technique was used to generate a short *KanMX* sequence flanked by sequences immediately up- and downstream of the open-reading frame to be deleted. These constructs were then transformed into cells to create the deletion strains of interest. The *KanMX* cassette confers geneticin (G418) resistance for selection.

Plasmid pAM7904, containing an HA-tagged allele of *TRK1*, was constructed by first amplifying the *TRK1* locus from wild-type genomic DNA [prepared as described (Burns *et al.*, 1996)] using oligonucleotides GT127 and GT128 (Table 1), both of which contain *Bam*HI sites. The PCR fragment was digested with *Bam*HI, followed by ligation into YEp352 (Hill *et al.*, 1986) to create pAW4. An *AflII* site approximately 60 bp from the 3′ end of the open-reading frame was used to insert a fragment encoding 3 HA tags in frame, created using oligonucleotides GT179 and GT180. Correct insertion was confirmed by *Sac*I digestion and by PCR using oligonucleotides GT178 and GT106, which bind upstream and downstream, respectively, of the insertion. pAM7904 complemented the hygromycin B sensitive phenotype of a *trk1Δ* strain as well as pAW4, demonstrating the tagged allele was functional. Expression of the HA tag was detected by Western blot analysis only in strains transformed with pAM7904, but not in strains transformed with pAW4 (containing untagged *TRK1*) or YEp352 (empty vector) (data not shown). Plasmids were transformed into strains of interest (Ito *et al.*, 1983) with heat shock times extended up to 2 hr.

**Table 1:** Oligonucleotides used in this study

GT106 aaaatccttttaatgcttaattaccttcttaaatattgagtacgaaaacctatttctaaagaatgagtatatatggaattcgagctcgtttaaac
GT127 ggcccgggatccgctttcctttcgcccattgt
GT128 ggcccgggatcccggttgtctttaagggaccg
GT178 ccttgagggcatgaaattgaag
GT179 cttaagtacccatacgatgttcctgactatgcgtacccatacgatgttcctgactatgcgtacccatacgatgttcctgactatgcgcttaaga
GT180 cttaagcgcatagtcaggaacatcgtatgggtacgcatagtcaggaacatcgtatgggtacgcatagtcaggaacatcgtatgggtacttaaga

Oligonucleotides GT127 and GT128 were used to amplify then clone *TRK1* into YEp352 (Hill *et al.*, 1986) to construct plasmid pAW4. Underlined regions are the *Bam*HI sites used for cloning. GT179 and GT180 contain 3 HA tags and were used to insert the tags near the 3′end of the *TRK1* open-reading frame. Correct insertion was verified using GT106 and GT178.

### Media

Media were prepared according to established procedures (Adams *et al.*, 1997, Sherman *et al.*, 1974). Cells were grown in rich YPD medium (1% yeast extract, 2% dextrose, 2% peptone) or YPAD medium (YPD with the addition of 40 mg/L adenine sulfate) or minimal SD medium (0.15% yeast nitrogen base without amino acids or ammonium sulfate, 0.5% ammonium sulfate, 2% dextrose) with necessary supplements to cover auxotrophies. For solid media, 2% agar was added. Heat-sensitive components, such as hygromycin B, were added after autoclaving, when the media had cooled to approximately 50°C. Dextrose, ammonium sulfate, yeast extract, peptone, yeast nitrogen base, and agar were from Fisher (Pittsburgh, PA). Amino acid and base supplements, KCl, hygromycin B, and sorbitol were from Sigma-Aldrich (St. Louis, MO).

### Hygromycin B sensitivity screen

**Figure 1:**
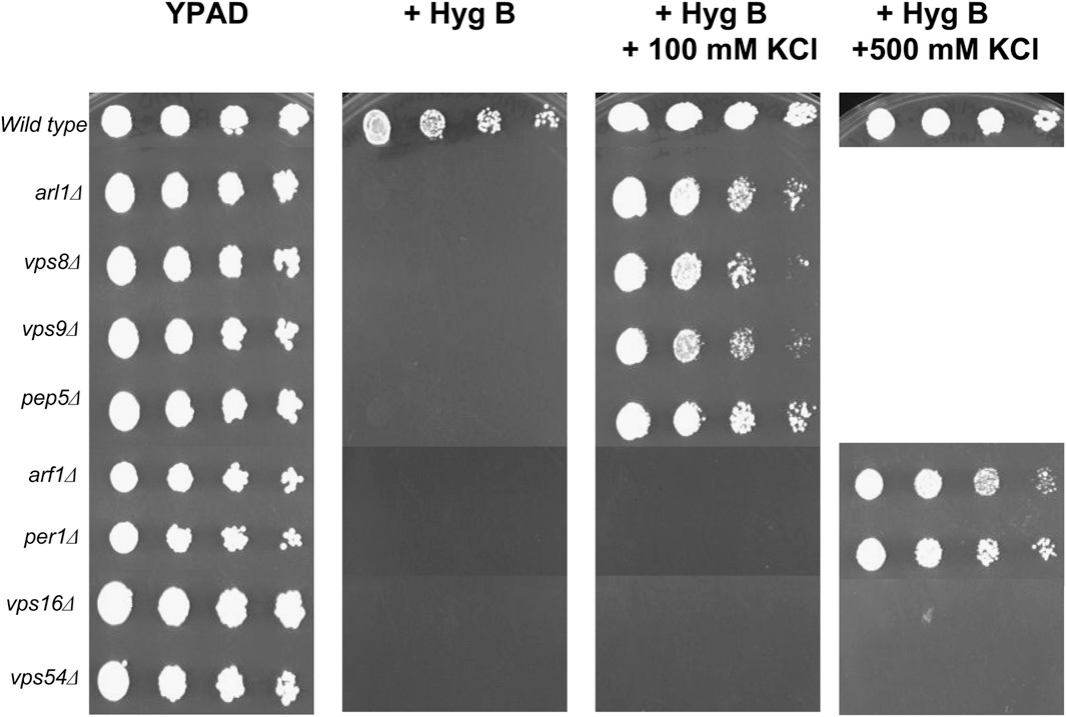
Representative data showing the three classes of hygromcyin B-sensitive mutants. The homozygous diploid deletion collection of 4653 strains was screened for sensitivity to hygromycin B in the presence or absence of added K^+^ (100 mm or 500 mm). Some strains displayed drug sensitivity that was suppressed by 100 mm KCl (including *arl1∆*, *vps8∆*, *vps9∆*, and *pep5∆*), others required 500 mm KCl for sensitivity to be suppressed (including *arf1∆* and *per1∆*), and others were not suppressed at all by the inclusion of KCl (including *vps16∆* and *vps54∆*). For the complete data set, see Table S1A, Table S1B, and Table S1C. YPAD = normal rich growth medium, + Hyg B = with the addition of 0.075 mg/ml hygromycin B with no addition, or with the addition of 100 mm KCl or 500 mm KCl. The indicated strains were grown overnight in YPAD medium, then diluted to 1.0 OD_600_/ml and subjected to 1:10 serial dilutions, spotted onto the indicated plates with a replicator tool, then grown for 2 days at 30°C. The total data set is reported in Table S1A, Table S1B, and Table S1C.

The 4653 deletion strains were originally obtained in 96-well microtiter plates. The strains in each plate were spotted onto solid YPD medium, then replica printed onto YPD medium with or without 0.1 mg/mL hygromycin B for the primary screen. Strains showing hygromycin B sensitivity were selected and subjected to a second round of screening as follows. Selected strains were grown in YPD at 30°C to saturation. Concentrations were standardized to an OD_600_ of 1.0, and each sample was 10-fold serially diluted and spotted with a replicator tool onto solid YPD medium with or without 0.075 mg/ml hygromycin B and with or without 100 or 500 mm KCl. Growth was scored after 4 days of incubation at 30°C. Typical results are shown in Figure 1; the complete data set is shown in supporting information, Table S1A, Table S1B, and Table S1C.

### ^86^Rb^+^ uptake assay

**Figure 2:**
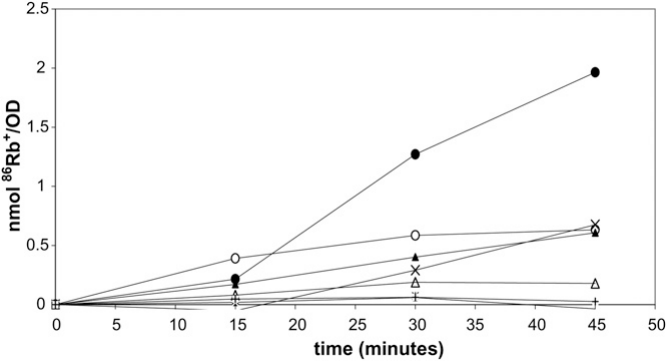
Representative data showing ^86^Rb^+^ uptake. Examples of data taken from the ^86^Rb^+^ uptake assay. Most membrane traffic mutants tested showed low levels of ^86^Rb^+^ uptake (*arl1Δ*, ▴ = *vps8Δ*, x = *vps9Δ*, ∆ = *vps21Δ*, and + = *mon2Δ*. Each strain was tested in triplicate on at least two different occasions. The data are summarized for the class I membrane traffic strains in Table 3. The total data set for all the membrane traffic mutants from all 3 classes is shown in Table S3.

Selected strains were tested for their ability to take up ^86^Rb^+^, a homolog of K^+^. Triplicate measurements of ^86^Rb^+^ uptake were performed as previously described (Mulet *et al.*, 1999, Manlandro *et al.*, 2005, Munson *et al.*, 2004a). Briefly, cells were grown to log phase in SD medium with appropriate supplements and 200 mm KCl. Five ODs were harvested, washed three times in distilled water, resuspended in 500 μL reaction medium (50 mm succinic acid, pH 5.5, 4% glucose). ^86^RbCl (1.1 μCi, 0.2 mm final concentration; PerkinElmer Life Sciences, Boston, MA) was added to each sample and 100 μL aliquots were taken over time, then diluted into 10 mL ice cold 20 mm MgCl_2_ to halt uptake. Cells were filtered over nitrocellulose membranes, washed three times with ice-cold 20 mm MgCl_2_ (10 mL each wash), and filter-bound radioactivity was measured by scintillation counting in UltimaGold XR scintillation fluid (Perkin-Elmer, Waltham, MA) using an LS 3801 scintillation counter (Beckman, Brea, CA). Uptake by each strain was measured on at least two different days; in each experiment, the wild-type strain was tested and average uptake was set to 100%. Typical results are shown in Figure 2; the complete data set is shown in Table S3. To measure ^86^Rb^+^ efflux, cells were incubated with ^86^RbCl for 60 min, then pelleted and resuspended in medium containing 0.2 mm (cold) RbCl. Aliquots of the cell suspension were removed over time and washed on filters as above.

### Carboxypeptidase Y (CPY) secretion assay

CPY secretion was performed as previously described (Roberts *et al.*, 1991). Briefly, cells were grown overnight in liquid YPAD medium, standardized to an OD_600_ of 1.0, serially diluted 10-fold, and spotted onto solid YPAD, YPAD with 500 mm KCl, and YPAD with 1 M sorbitol. Cells were allowed to grow for 24 hr, then a nitrocellulose membrane (Schleicher & Schuell, Keene, NH or Whatman, GE Healthcare, Piscataway, NJ) was laid over the cells and growth continued for another 24 hr. Membranes were washed with dH_2_O to remove remaining cells, then incubated for 30 min in PBS-T (9% NaCl, 0.14% KH_2_PO_4_, 0.42% NaHPO_4_, 0.02% Tween 20, pH 7.4) and 30 min in blocking buffer (5% Carnation instant milk in PBS-T) with gentle rocking at room temperature. Membranes were incubated in a 1:2000 dilution of mouse anti-CPY monoclonal antibody (Molecular Probes/InVitrogen, Carlsbad, CA) for 1 hr with shaking at room temperature. Membranes were washed three times in blocking buffer and incubated in a 1:2000 dilution of rabbit anti-mouse IgG-horseradish peroxidase conjugated secondary antibody (Amersham Biosciences, GE Healthcare) for 1 hr. Membranes were washed three times in PBS-T and CPY antigen was visualized using ECL western blotting detection reagent (Amersham Biosciences, GE Healthcare) according to the manufacturer’s instructions using Kodak X-Omat AR film (Rochester, NY). Exposure time for visualization of luminescence was up to 10 min. As a control for lysis, parallel filters were subjected to similar analysis with an anti-phosphoglycerol kinase (PGK) antibody (Molecular Probes/InVitrogen).

### Subcellular fractionation and raft analysis

To examine localization of Trk1 in the various mutant strains, 100 ODs of cells previously transformed with pAM7904 (HA-tagged *TRK1*) were collected by centrifugation then washed once with breaking buffer (10 mm Tris-HCl, pH 7.4, 0.3 M sorbitol, 0.1 M NaCl, and 5 mm MgCl_2_), then incubated for 1 hr on ice in 1 mL breaking buffer containing protease inhibitors (20 μg/mL phenylmethylsulfonyl fluoride, 1 μg/mL antipain, 1 μg/mL leupeptin, 1 μg/mL pepstatin, and 10 μg/mL α_2_-macroglobulin, all from Sigma-Aldrich) and phosphatase inhibitors (10 mm NaF, 5 mm β-glycerophosphate, and 1 mm NaVO_4_, also all from Sigma-Aldrich). Samples were then transferred to 13 × 100 mm glass tubes containing the same volume of acid-washed glass beads. Samples were vortexed for 1 min four times, with 1 min on ice in between each vortexing. The glass tubes were spun briefly at low speed to pellet the glass beads, then lysates were transferred to microcentrifuge tubes. Lysates were centrifuged first at 700 × *g*, then at 1000 × *g* to remove remaining beads and large debris. The cleared supernatants, with additional aliquots of protease and phosphatase inhibitors, were used either for differential centrifugation or for raft analysis. Previously, we found that in wild-type cells, Trk1 is pelleted by centrifugation at 13,000 × *g*, and that none is found in the 13,000 × *g* supernatant fraction, consistent with plasma membrane localization of Trk1 (Munson *et al.*, 2004a). Thus, we analyzed the distribution of Trk1 between the 13,000 × *g* pellet and supernatant fractions.

Analysis of incorporation of Trk1-HA into lipid microdomains (“rafts”) was determined as previously described (Zeng *et al.*, 2004). Cleared lysates were treated with an equal volume of cold 2% Triton X-100 and incubated on ice for 30 min. Pellet (containing the raft proteins) and supernatant fractions were obtained after centrifugation at 100,000 × *g* for 1 hr at 4°C.

Pellets were resuspended in 1× Laemmli sample buffer (Laemmli 1970) and supernatant samples were brought to 1× by the addition of concentrated sample buffer. Samples were separated by gel electrophoresis on 4–15% gradient gels (Bio-Rad, Hercules, CA) then transferred to nitrocellulose. Western blot analysis with an anti-HA antibody (Covance, Princeton, NJ) was performed as previously described (Ausubel *et al.*, 1997). As a control, samples were also blotted with the anti-PGK antibody (Molecular Probes/InVitrogen).

### Gene ontology (GO) term analysis

The *Saccharomyces* Genome Database (SGD) (http://www.yeastgenome.org; accessed between June and August 2010) was used to identify GO terms for process, function, and component. The GO Term Finder function (Boyle *et al.*, 2004) using the default settings which includes noncoding genes was used to calculate *P* values describing enrichment of GO terms in the selected set of hygromycin B-sensitive mutants compared to the 7167 gene elements in the yeast genome. The significance cutoff is set to return *P* values < 0.01. A subset of the data from this analysis with the most significant hits is shown in Table 2; the complete GO analysis is documented in Table S2A, Table S2B, and Table S2C.

**Table 2:** Top 5 gene ontology (GO) terms

GO Term	Cluster Frequency (%)	Background Frequency (%)	Fold Enrichment	Significance (*P* Value)
A. Process terms (Complete set of 156 genes)				
Vesicle-mediated transport	32.7	5.0	6.5	2.34E-26
Endosome transport	19.3	0.9	21.4	5.27E-26
Vacuolar transport	21.2	1.8	11.8	1.88E-24
Cellular localization	42.3	10.4	4.1	7.59E-23
Intracellular transport	36.5	8.3	4.4	9.74E-21
B. Function terms (Complete set of 156 genes)				
Protein binding	27.6	8.6	3.2	2.58E-10
Phosphoinositide binding	7.7	0.8	9.6	3.36E-07
Phospholipid binding	7.7	0.9	8.6	2.27E-06
Lipid binding	7.7	1.0	7.7	6.13E-06
Ubiquitin binding	4.5	0.4	11.3	1.10E-04
C. Component terms (complete set of 156 genes)				
Endosome	17.3	1.4	12.4	2.65E-20
Endosomal part	10.3	0.4	25.8	8.62E-17
Golgi apparatus	19.2	2.5	7.7	1.64E-16
Golgi apparatus part	16.0	1.8	8.9	3.71E-15
Protein complex	46.8	18.0	2.6	1.06E-14
D. Process terms (76 class I genes)				
Vesicle-mediated transport	38.2	5.0	7.6	1.69E-16
Endosome transport	21.1	0.9	23.4	7.15E-16
Cellular localization	50.0	10.4	4.8	1.04E-15
Intracellular transport	44.7	8.3	5.4	4.07E-15
Vacuolar transport	25.0	1.8	13.9	8.73E-15
E. Process terms (23 class II genes)				
Post-Golgi vesicle-mediated transport	21.7	1.0	21.7	2.90E-04
Vacuolar transport	26.1	1.8	14.5	2.90E-04
Intracellular transport	43.5	8.3	5.2	7.90E-04
Establishment of localization in cell	43.5	9.0	4.8	1.67E-03
Organelle inheritance	17.4	0.7	24.9	2.54E-03
F. Process terms (57 class III genes)				
Vesicle-mediated transport	26.3	5.0	5.3	2.32E-05
Biological regulation	45.6	16.9	2.7	1.40E-04
Endosome transport	12.3	0.9	13.7	2.30E-04
Post-Golgi vesicle-mediated transport	12.3	1.0	12.3	3.90E-04
Golgi vesicle transport	17.5	2.5	7.0	4.00E-04

The entire set of 156 hits from the hygromycin B sensitivity screen was submitted to GO Term Finder (Boyle *et al.*, 2004) on the SGD (http://www.yeastgenome.org, accessed August 2010). Using the default settings, the 156 genes were analyzed for enrichment of process terms (A), function terms (B), and component terms (C) compared to the background set of 7167 genes in the yeast genome. Similar analysis for process terms was performed for the genes falling into class I (D), class II (E), and class III (F). The top five terms as given by lowest *P* value are shown, as well as the frequency of that term in the cluster of genes in the group (either the complete set of 156, the 76 class I genes, the 23 class II genes, or the 57 class III genes), compared to the frequency in the complete set of genes in the genome. The complete analyses for the entire set of genes (corresponding to A, B, and C) are found in Table S2B and the analyses for the process terms for the individual classes (corresponding to D, E, and F) are found in Table S2C.

## Results and Discussion

Regulation of cytosolic K^+^ levels, controlled by the combined activities of influx, efflux, and sequestration, is not well understood despite the fact that K^+^ is the most abundant cation in cells. Processes that require K^+^ include protein synthesis (Ledbetter and Lubin, 1977, Lubin, 1967, Lubin and Ennis, 1964, Lubin 1964) and progression through the cell cycle (Sutton *et al.*, 1991, Masuda *et al.*, 2000). Endocytosis is also affected by intracellular K^+^ (Larkin *et al.*, 1983, Larkin *et al.*, 1986, Heuser and Anderson, 1989, Larkin *et al.*, 1985, Vercauteren *et al.*, 2010). In efforts to identify gene products that contribute to control of K^+^ levels, we screened the yeast knockout collection.

### A small fraction of the yeast deletion collection is sensitive to hygromycin B

Our rationale was as follows: Anecdotal reports in the literature suggest that sensitivity to toxic cations, including hygromycin B, is a characteristic of strains exhibiting alterations in K^+^ homeostasis. Examples include *trkΔ* mutants, lacking the K^+^ influx transporters, Trk1 and Trk2 (Madrid *et al.*, 1998); *halΔ* mutants, lacking the kinases Sat4/Hal4 and Hal5, which function upstream of the Trks (Mulet *et al.*, 1999); calcineurin mutants, lacking an important phosphatase for regulation of intracellular levels of ions, including K^+^ (Withee *et al.*, 1998); and *pmp3Δ* mutants, lacking a small proteolipid important for cellular responses to ions (Navarre and Goffeau 2000). Thus, we hypothesized that mutants sensitive to hygromycin B would likely have defects in K^+^ regulation.

We identified potential K^+^ homeostasis mutants by screening the yeast homozygous diploid deletion collection, which represents about two-thirds of the genome and includes all nonessential genes, for sensitivity to hygromycin B. Although hygromycin B is a translation inhibitor (Brodersen *et al.*, 2000), ribosomal protein genes are for the most part essential and so not present in this collection. In addition, although some glycosylation mutants are sensitive to hygromycin B (Sato *et al.*, 1999, Dean, 1995, Van Berkel *et al.*, 1999), such mutants are generally sensitive to much higher levels of hygromycin B than the levels we used here. Rather, it has been suggested hygromycin B at lower levels like those used for the screen is useful for identification of mutant strains with defects in stress tolerance (Banuelos *et al.*, 2010, Dudley *et al.*, 2005), including ion misregulation. Thus, it seemed reasonable to expect that many of the mutants identified by our screen would be involved in K^+^ homeostasis, rather than translation or glycosylation.

The collection was initially screened to identify mutant strains that grew slowly in the presence of hygromycin B as described in Materials and Methods. Strains that passed the primary screen were rescreened to verify the hygromycin B-sensitive phenotype. A total of 166 mutants (∼3.5% of the collection) were identified. As expected, few of the mutants obtained were deleted for genes involved in glycosylation or translation. Ten of the mutants had deletions of so-called “dubious” open-reading frames (ORFs) according to the SGD and were not analyzed further, although we noted that for the most part, these dubious genes overlapped authentic genes identified in the screen. The remaining 156 strains, as previously detailed in our studies on the *arl1Δ* mutant (Munson *et al.*, 2004b), were also sensitive to tetramethylammonium chloride (data not shown), demonstrating a general sensitivity to toxic cations, rather than hygromycin B in particular.

The mutants obtained were further subdivided by the ability of KCl in the growth medium to suppress hygromycin B sensitivity, since we previously noted that excess K^+^ could suppress the sensitivity of the *arl1Δ* strain (Munson *et al.*, 2004a). Seventy-six of the 156 strains (49% of the sensitive mutants) grew well in hygromycin B-medium supplemented with 100 mm KCl. Twenty-three strains (15% of the sensitive mutants) grew in hygromycin B-medium supplemented with 500 mm KCl. Fifty-seven strains (36% of the sensitive mutants) did not achieve levels of growth seen in normal rich medium even with the inclusion of 500 mm KCl to hygromycin B-medium. These three groups were named class I, II, and III, respectively. Examples are shown in Figure 1. The ability of K^+^ to reverse the hygromycin B sensitivity of these strains was specific, since inclusion of an equal osmolar concentration of sorbitol was unable to suppress sensitivity to the drug (data not shown). The complete data sets from the screen are shown in Table S1A, Table S1B, and Table S1C. As an internal validation, we re-isolated the *arl1Δ* mutant in the screen, but in addition, isolated the *trk1Δ*, *sat4/hal4Δ*, *hal5Δ*, and *cnb1Δ* strains; sensitivity of these strains was suppressed by 100 mm KCl, placing them in class I. (*CNB1* encodes the regulatory subunit of calcineurin (Cyert *et al.*, 1991). In contrast, the catalytic subunit of calcinuerin is encoded by one of two paralogs, *CNA1* or *CNA2* (Cyert *et al.*, 1991), and one is sufficient to confer calcineurin activity on cells.) Finally, the *pmp3Δ* strain was isolated as well—its sensitivity could not be suppressed by 500 mm KCl, making it a member of class III. Comparing our results to those previously obtained demonstrated that although the SGD (http://www.yeastgenome.org) lists 359 unique gene entries, we obtained only 156 hits. However, while we clearly did not obtain all the genes on the SGD list (121of 156 entries in our list are found on the SGD list, 77%), we did identify a number of genes that are not currently present on the SGD list (38 entries or 23% of our list). These identifications are noted in Table S2A—upper case locus and gene names denote genes previously identified according to SGD (accessed August 2010), while lower case locus and gene names denote those newly identified by our screen. Some of the differences might be due to different strain backgrounds or differences in environmental conditions, including the source of hygromycin B, which we have found to vary from lot to lot.

### A large portion of the deletion mutant strains are membrane traffic mutants

We organized the strains obtained from the screen by function of the deleted gene according to the gene ontology (GO) information from SGD (Table S2A). The most abundant group as a whole has deletions in genes previously shown to be important for regulation of membrane traffic (55 or 35% of the 156 strains). A similar percentage of genes on the SGD list of hygromycin B-sensitive strains are also membrane traffic regulators. Analysis of the GO terms by enrichment was performed for the entire set of 156 genes for process, function, and component terms (Table S2B) and for each of the genes found in the three classes for process terms only (Table S2C). The top 5 hits in terms of significance (as measured by *P* value) for each of these analyses is shown in Table 2. The major conclusion is that genes encoding regulators of membrane traffic are highly enriched in the collection of mutants isolated by our screen.

Of the genes we identified, many have been shown to encode members of protein complexes. However, not all members of each complex were obtained in our screen. For example, the endosomal sorting complex required for transport (ESCRT) complexes have important roles in sorting cargo into the multivesicular body (MVB) pathway. All of the ESCRT-related strains obtained in the initial screen were members of class I, and we obtained at least one member from each of the complexes. It is possible that conditions in the screen were not sufficiently stringent to identify all members of each complex. This possibility seemed likely given that we failed to identify known hygromycin B-sensitive mutants according to SGD. Alternatively, it is possible that not all of these gene products are required for regulation of intracellular K^+^ content and therefore their loss does not confer a hygromycin B phenotype. We therefore specifically examined strains with deletions of genes known to encode MVB-pathway proteins not obtained in the initial screen. Our results demonstrated that several of the ESCRT mutants not obtained in the initial screen were sensitive to hygromycin B, including *snf8Δ/vps22Δ* and *vps25Δ* (both members of ESCRT-II), *vps28Δ* (ESCRT-I), and *snf7Δ/vps32Δ* (ESCRT-III), consistent with data from SGD. All of these mutants were suppressed by 100 mm KCl, placing them in class I. However, several mutants showed little sensitivity to hygromycin B at the levels tested (up to 0.1 mg/ml), including *hse1Δ* (ESCRT-0), *srn2Δ/vps37Δ* (ESCRT-I), and strains missing genes encoding MVB accessory factors (*doa4Δ*, *rim13Δ*, or *rim20Δ*). In accordance with our results, none of these genes are listed in the SGD list of gene mutations conferring a hygromycin B-sensitive phenotype.

We also obtained members of other complexes involved in membrane traffic, but the phenotypes of the mutants within the complex were not always the same. First, we obtained the strains missing each of the four members of the Golgi-associated retrograde protein (GARP/VFT) complex, *vps51Δ*, *sac2Δ/vps52Δ*, *vps53Δ*, and *luv1Δ/vps54Δ* previously reported to be sensitive to hygromycin B (Conboy and Cyert 2000). However, *vps52Δ* was a member of class II, while the other three strains were members of class III. Second, we obtained several strains missing a member of the class C core vacuole/endosome tethering (CORVET) and the related homotypic fusion and protein sorting (HOPS) complexes (Nickerson *et al.*, 2009), including the *vps3Δ* (class II), *vps8Δ* (class I), *pep5Δ/vps11Δ* (class I), *vps16Δ* (class III), *vps33Δ* (class III), and *vps41Δ* (class I) strains. We also obtained *vps21Δ* (class I), missing the gene for the Rab protein that associates with HOPS/CORVET complexes and mediates vesicle fusion (Gerrard *et al.*, 2000, Horazdovsky *et al.*, 1994), and *vps9Δ* (class I), missing the gene for the guanine nucleotide exchange factor that activates Vps21 (Hama *et al.*, 1999). We did not obtain the *vps18Δ* or *vps39Δ* strains, neither of which have been previously found to be sensitive to hygromycin B according to SGD. Taken as a whole, these results suggest that for the most part, loss of different subunits in a particular complex results in similar but not identical phenotypes with respect to hygromycin B-sensitivity and suppression by added K^+^.

It has been suggested that changes in polarity of the plasma membrane may be responsible for hygromycin B sensitivity. Hyperpolarization thus leads to increased uptake of toxic cations, which can be reversed by the addition of an excess of a nontoxic cation, in this case K^+^. This hypothesis was put forward by Perlin and colleagues (Perlin *et al.*, 1988, Perlin *et al.*, 1989) with respect to their studies on the plasma membrane H^+^-ATPase, Pma1. This hypothesis could explain the hygromycin B-sensitive phenotype of the class I mutants, which have phenotypes similar to the *trk1Δ* mutant, since hygromycin B sensitivity was reversed by the addition of 100 mm K^+^. However, other work has shown that other mutants, such as *kha1Δ* mutants, exhibit normal polarity but nevertheless show hygromycin B sensitivity (Maresova *et al.*, 2006). This fact is consistent with our class III mutants, where we found that a >250 molar excess of K^+^ was unable to suppress hygromycin B sensitivity.

### Most membrane traffic mutants exhibit defective K^+^ influx

While our results thus far suggest that sensitivity to hygromycin B is a useful screening tool, we wanted to establish more directly that the mutants obtained by the screen were defective in K^+^ homeostasis. We therefore investigated the ability of the hygromycin B-sensitive strains to take up ^86^Rb^+^ relative to the parental wild-type strain. ^86^Rb^+^ has been used extensively to measure K^+^ fluxes in *Saccharomyces* (Madrid *et al.*, 1998, Mulet *et al.*, 1999, Banuelos and Rodriguez-Navarro 1998) and has been shown to be fully competitive with K^+^ (Armstrong and Rothstein 1967).

For this analysis, we examined only the membrane traffic mutant strains obtained from the initial screen. Examples are shown in Figure 2 (for the complete list, as well as relative levels compared to wild-type, see Table S3). Most of the strains exhibited decreased ^86^Rb^+^ influx (<75% of wild-type levels), including 81% of class I membrane traffic mutants (25 of 30 strains), 92% of class II membrane traffic mutants (11 of 12 strains), and 60% of class III membrane traffic mutants (six of 10 strains tested; four were not tested). Most of the remaining strains took up levels of ^86^Rb^+^ close to those exhibited by wild-type (considered to be those exhibiting 75–125% of wild-type levels). Four strains exhibited more than 125% of wild-type uptake: *rer1Δ* (class I; 151% of wild-type); *tlg2Δ* (class III; 276% of wild-type); *vps51Δ* (class III; 372% of wild-type); and *vps53Δ* (class III; 142% of wild-type). In other work, we have found that some strains with increased K^+^ efflux take up higher levels of ^86^Rb^+^, presumably as a compensatory mechanism to maintain intracellular levels of K^+^ (Manlandro *et al.*, 2005). To test this, these four strains were loaded with ^86^Rb^+^ for 60 min, then washed and resuspended in buffer containing 20 mm (cold) RbCl. Two strains, *vps51Δ* and *vps53Δ* (these genes encode members of the GARP/VFT complex), showed increased efflux compared to wild-type (2-threefold faster). The other two strains with increased uptake, *rer1Δ* and *tlg2Δ*, exhibited efflux rates similar to wild-type (data not shown). Taken as a whole, the ^86^Rb^+^ uptake experiments demonstrate that while the hygromycin B-sensitive phenotype is a useful primary screen, it does not always correlate with decreased K^+^ (Rb^+^) uptake.

In summary, as shown in Figure 2 and Table S3, the vast majority of the hygromycin B-sensitive membrane traffic mutants were defective for uptake of ^86^Rb^+^, a more specific test of K^+^ defects than hygromycin B sensitivity. Thus, the hygromycin B screen provided a valid approach to identify mutants with disturbances in K^+^ uptake compared to wild-type cells.

### Most membrane traffic mutants localize Trk1 correctly

As discussed above, loss of specific genes encoding membrane traffic regulators lead to decreased influx of K^+^ in most cases. We hypothesized that these strains could be defective for delivery and/or retention of the major K^+^ transporter, Trk1, at the plasma membrane because of the membrane traffic defects, although our previous studies demonstrated that this was not the case for the *arl1Δ* mutant (Munson *et al.*, 2004a). We confined the next set of studies to 29 of the 30 membrane traffic mutants from class I, since the hygromycin B sensitive phenotype of these strains, like that of the *trk1Δ* and *arl1Δ* strains, can be suppressed by 100 mm KCl. One of these 29 strains, *rer1Δ*, had increased influx and WT efflux of K^+^ (^86^Rb^+^); five strains (*cog6Δ*, *gga1Δ*, *gga2Δ*, *pep5Δ*, and *vps27Δ*) had influx rates similar to wild-type; but the remaining 23 strains had influx rates less than wild-type.

We first asked whether overexpression of Trk1, the major K^+^ influx transporter, suppressed the hygromycin B-sensitive phenotype of these strains. The strains were transformed with either a high copy empty vector (YEp352) or plasmid pAM7904, bearing an HA-tagged allele of *TRK1* in the same vector. The tagged allele was functional because it complemented the hygromycin B-sensitive phenotype of the *trk1Δ* strain. Transformants were selected on medium lacking uracil then tested on YPAD medium with or without the addition of hygromycin B. However, none of the mutants grew better on medium containing hygromycin B when transformed with *TRK1-HA* compared to empty vector. Therefore, simply expressing higher levels of Trk1 was not sufficient to abrogate the hygromycin B-sensitive phenotype in any of the strains.

**Figure 3:**
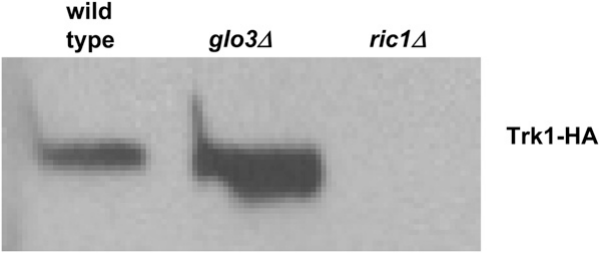
Steady state levels of Trk1-HA in representative membrane traffic mutant strains. Wild-type (BY4743) and mutant strains were transformed with a 2 μ plasmid containing *TRK1-HA*. Cells were grown, then lysates were prepared for Western blot analysis with an anti-HA primary antibody followed by an HRP-conjugated secondary as described in Materials and Methods. Equal numbers of cells were prepared for each sample. The majority of strains tested showed levels of Trk1-HA similar to that of wild-type. Here we show one strain, *glo3Δ*, which overexpresses Trk1-HA, and one strain, *ric1Δ*, which lacks expression of Trk1-HA. Each of the 29 transformed class I membrane traffic mutant strains was tested at least twice, and the data are summarized in Table 3.

To determine which genes were responsible for stability and/or localization of Trk1, we examined Trk1-HA in the class I membrane traffic mutants. In the first set of experiments, lysates were prepared, separated by electrophoresis, blotted to nitrocellulose, then probed with an anti-HA antibody to determine relative levels of Trk1. Two of the 29 showed no Trk1-HA: *bro1Δ* and *ric1Δ*. An example is shown in Figure 3; data are summarized in Table 3, column 5. Bro1 is an accessory factor for the MVB pathway (Springael *et al.*, 2002). It is interesting to note that loss of *RIC1* has a different phenotype compared to loss of *RGP1* with respect to steady state levels of Trk1, despite the fact their gene products work together as the guanine nucleotide exchange factor for Ypt6 (Siniossoglou *et al.*, 2000). In addition, in the *bro1Δ* and *ric1Δ* strains, although unable to detect the tagged version of Trk1 by western blot analysis, we nevertheless detected some ^86^Rb^+^ uptake, especially in the *ric1Δ* strain, suggesting that the cells may respond by up-regulating other means of K^+^ uptake (*i.e.*, Trk2 or less specific monovalent cation influx transporters), although we cannot rule out the possibility that the native untagged allele is expressed.

**Table 3:** Class I membrane traffic mutants: summary of K^+^ and Trk1-HA phenotypes

Strain	ORF	Protein Function (Ref)	1	2	3	4	5	6	7
			YPAD	+HB	+HB +K^+^	^86^Rb^+^ uptake	Trk1 Level	% in P13	% in Rafts
Wild-type	—	—	++++	+++	++++	100%	WT	100%	100%
*trk1Δ*	YJL129C	K^+^ transport (Gaber *et al.* 1988)	++++	—	++++	10%	n.d.	n.d.	n.d.
*arl1Δ*	YBR164C	Arf-like (Rosenwald *et al.* 2002)	++++	—	++++	68%	WT	100%	100%
*bro1Δ*	YPL084W	MVB path (Katzmann *et al.* 2002)	++++	—	++++	8%	*	*	*
*chs5Δ*	YLR330W	Chs3 local. (Santos *et al.* 1997)	++++	—	++++	33%	WT	100%	100%
*cog5Δ*	YNL051W	COG complex (Oka and Krieger 2005)	++++	+/−	++++	67%	WT	100%	100%
*cog6Δ*	YNL041C	COG complex (Oka and Krieger 2005)	++++	+	++++	116%	WT	100%	∼90%
*did4Δ*	YKL002W	ESCRT-III (Katzmann *et al.* 2002)	++++	—	++++	62%	WT	100%	100%
*gga1Δ*	YDR358W	Golgi γ adaptin (Zhdankina *et al.* 2001)	++++	+	++++	81%	WT	100%	100%
*gga2Δ*	YHR108W	Golgi γ adaptin (Zhdankina *et al.* 2000)	++++	—	++++	122%	WT	100%	100%
*glo3Δ*	YER122C	Arf GAP (Poon *et al.* 1999)	+++	—	+++	41%	>WT	∼75%	∼75%
*gos1Δ*	YHL031C	v-SNARE (Bensen *et al.* 2001)	++++	+/−	+++	38%	n.d.	n.d.	n.d.
*mon2Δ*	YNL297C	Regulator of Arl1 nucleotide binding (Jochum *et al.* 2002)	++++	+/−	+++	34%	WT	100%	100%
*pep5Δ*	YMR231W	Vac Fusion (Robinson *et al.* 1988)	++++	—	++++	76%	WT	100%	100%
*rer1Δ*	YCL001W	ER retention (Nishikawa and Nakano 1993)	++++	—	+++	151%	WT	100%	100%
*rgp1Δ*	YDR137W	Ypt6 GEF (Siniossoglou *et al.* 2000)	++++	+/−	++++	56%	WT	100%	100%
*ric1Δ*	YLR039C	Ypt6 GEF (Siniossoglou *et al.* 2000)	++++	+	++++	47%	*	*	*
*sec22Δ*	YLR268W	R-SNARE (Gaynor and Emr 1997)	++++	—	+++	49%	WT	∼75%	∼75%
*vam3Δ*	YOR106W	T-SNARE (Burri and Lithgow 2004)	++++	—	++++	75%	WT	100%	100%
*vam7Δ*	YGL212W	V-SNARE (Burri and Lithgow 2004)	++++	—	++++	39%	WT	100%	100%
*vps4Δ*	YPR173C	AAA-ATPase (Katzmann *et al.* 2002)	++++	—	++++	55%	WT	100%	100%
*vps8Δ*	YAL002W	Binds Vps21 (Horazdovsky *et al.* 1996)	++++	—	++++	58%	WT	100%	100%
*vps9Δ*	YML097C	MVB Path (Katzmann *et al.* 2002)	++++	—	++++	61%	WT	100%	100%
*vps20Δ*	YMR077C	ESCRT-III (Katzmann *et al.* 2002)	++++	—	++++	75%	WT	100%	100%
*vps21Δ*	YOR089C	Rab (Horazdovsky *et al.* 1996)	+++	—	++++	56%	WT	100%	100%
*vps23Δ*	YCL008C	ESCRT-I (Katzmann *et al.* 2002)	++++	—	++++	13%	WT	100%	∼75%
*vps24Δ*	YKL041W	ESCRT-III (Katzmann *et al.* 2002)	++++	—	++++	23%	WT	100%	100%
*vps27Δ*	YNR006W	MVB path (Katzmann *et al.* 2002)	++++	—	++++	90%	>WT	100%	∼75%
*vps30Δ*	YPL120W	Vac Sorting (Robinson *et al.* 1988)	++++	—	++++	47%	WT	100%	100%
*vps36Δ*	YLR417W	ESCRT-II (Katzmann *et al.* 2002)	++++	—	++++	29%	WT	100%	100%
*vps41Δ*	YDR080W	HOPS (Wang *et al.* 2003)	++++	—	+++	52%	WT	∼75%	100%
*ypt6Δ*	YLR262C	Rab (Li and Warner 1996)	++++	+/−	++++	54%	WT	100%	100%

**Figure 4:**
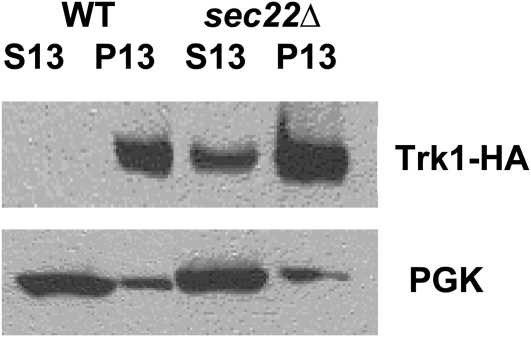
Subcellular fractionation analysis of representative strains. Wild-type (BY4743) and mutant strains transformed with 2 μ plasmid containing *TRK1-HA* were grown, then lysed and prepared for subcellular fractionation analysis as described in Materials and Methods. The majority of strains tested showed all Trk1-HA in the P13 fraction as for wild-type shown here, consistent with plasma membrane localization. However, a few strains, including *sec22Δ*, showed a portion of Trk1-HA in the S13 fraction, consistent with the notion that a portion of the protein was contained in an internal membrane pool. The small amount of PGK in the P fractions is due to incomplete removal of the supernatant in an effort to avoid disturbing the pellet. Each of the 29 class I transformed membrane traffic mutant strains was tested at least twice, and the data are summarized in Table 3.

**Figure 5:**
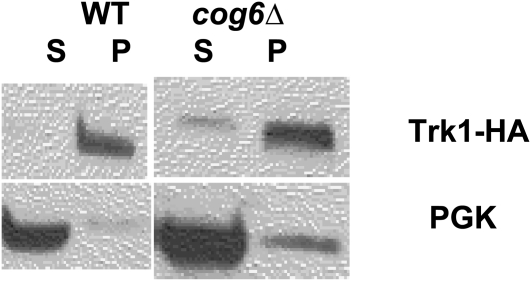
Trk1-HA localization to lipid microdomains in representative strains. Wild-type (BY4743) and mutant strains transformed with 2 μ plasmid containing *TRK1-HA* were grown and then were lysed, treated with Triton X-100, and prepared for raft analysis as described in Materials and Methods. T = total, S = supernatant from the 100,000 × *g* spin (Triton-X soluble fraction), and *P* = pellet from the 100,000 × *g* spin (the Triton-X insoluble fraction contains the lipid microdomains or rafts). The majority of strains tested localized Trk1-HA exclusively to the Triton-X 100 pellet as shown here for wild-type. However, a few strains, as shown here by *cog6Δ*, did not localize Trk1-HA exclusively to the raft fraction. Each of the 29 transformed class I membrane traffic mutant strains was tested at least twice and the data are summarized in Table 3.

Thirty membrane traffic mutants from the homozygous diploid deletion collection grow well in normal medium (YPAD; column 1) and are sensitive to hygromcyin B (0.075 mg/ml; column 2), but sensitivity is suppressed by the presence of 100 mm KCl, placing them in class I [column 3; KCl alone had no effect on growth (Table S1A)]. The phenotypes of the wild-type (WT) and *trk1Δ* strains are shown for comparison. Growth scale: –, no growth; +/−, weak growth; ++++, robust growth. The majority are defective for uptake of ^86^Rb^+^ compared to WT (column 4, measured at 30 min in triplicate as in Figure 2; data shown are the average of at least two determinations). For columns 5, 6, and 7, the strains were transformed with a high copy plasmid containing *TRK1-HA* and steady state levels (column 5, levels relative to WT as in Figure 3), subcellular localization (column 6, % Trk1-HA found in the P13 fraction as in Figure 4), and raft localization (column 7; % Trk1 found in TX-100 pellet fraction as in Figure 5) were determined. All experiments were performed at least twice with similar results. ^*^No expression of Trk1-HA. ND, not determined (despite multiple attempts using various approaches, we were unable to transform the *gos1Δ* strain with any vector).

Two other mutant strains showed increased levels of Trk1-HA, including *glo3Δ* and *vps27Δ* (an example is shown in Figure 3; data are summarized in Table 3, column 5). Glo3 is an Arf guanine nucleotide exchange factor (Poon *et al.*, 1999, Szafer *et al.*, 2001) and Vps27 is a member of the ESCRT-0 complex (Bilodeau *et al.*, 2002, Bilodeau *et al.*, 2003, Katzmann *et al.*, 2003). For the strains which show differences in the steady state levels of Trk1-HA, we have not explored whether this occurs as a result of different rates of synthesis or degradation of the protein. Importantly, however, the remaining 25 strains had levels of Trk1-HA equivalent to that observed in the wild-type parent.

We next determined whether the Trk1-HA in each of the mutant strains was localized correctly to the plasma membrane. Lysates were prepared and subjected to subcellular fractionation by differential centrifugation. Plasma membrane and vacuole fractions pellet at 13,000 × *g* (P13), while internal membranes (Golgi and endosomes) are present in the supernatant (S13), which would then pellet at 100,000 × *g* (P100) (Gaynor *et al.*, 1998). Since Trk1 is an integral membrane protein with eight membrane passes (Durell *et al.*, 1999, Durell and Guy 1999), we reasoned that determination of the fraction in P13 relative to S13 was sufficient to determine delivery of the Trk1-HA to the plasma membrane. Moreover, we previously showed that a myc-tagged allele of Trk1 was found in the P13 fraction, not in the P100 or S100 fraction in wild-type or *arl1Δ* cells (Munson *et al.*, 2004a). Three strains – *glo3Δ*, *sec22Δ*, and *vps41Δ* – reproducibly exhibited a moderate fraction of Trk1-HA in the P13 supernatant fraction suggestive of localization to an internal membrane pool (an example is shown Figure 4; data are summarized in Table 3, column 6). Sec22 is an R-SNARE (Gaynor and Emr 1997), while Vps41 is a member of the HOPS complex (Radisky *et al.*, 1997, Wurmser *et al.*, 2000). In the remaining 26 strains, however, like wild-type, all of the Trk1-HA was found in the P13 fraction, consistent with correct localization to the plasma membrane.

To examine localization of Trk1-HA to lipid microdomains, lysates were incubated with Triton-X 100 on ice for 30 min (Zeng *et al.*, 2004). The lysates were then separated by centrifugation at 100,000 × *g* into pellet (raft) and supernatant fractions. In wild-type cells, Trk1-HA is localized to the pellet fraction (Yenush *et al.*, 2005) as confirmed here. However, in five of the mutants, *cog6Δ*, *glo3Δ*, *sec22Δ*, *vps23Δ*, and *vps27Δ*, some Trk1-HA was localized to the supernatant fraction rather than the Triton-X 100 insoluble pellet (an example is shown in Figure 5; data are summarized in Table 3, column 7). However, in the remaining 24 strains, like wild-type, Trk1-HA was found only in the Triton-X 100 pellet, consistent with correct localization to lipid microdomains.

Thus, in the majority of mutant strains examined (21 of 29), including the *arl1Δ* mutant as previously found (Munson *et al.*, 2004a), neither steady-state level nor localization of Trk1-HA was significantly different from that seen in wild-type cells, and most of the remainder (six of eight) had relatively mild disruption of Trk1-HA location (*i.e.* where disruption was observed, the majority of the protein was nevertheless in the correct location). The major exceptions were the *bro1Δ/vps31Δ* and *ric1Δ* strains, both of which had undetectable levels of Trk1-HA.

### Effect of added K^+^ to strains exhibiting a CPY secretion phenotype

**Figure 6:**
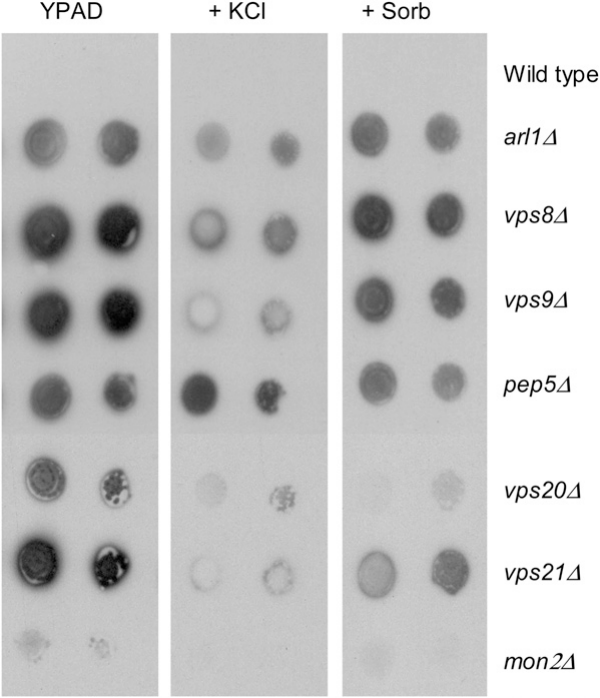
Effects of K^+^ on CPY secretion in selected membrane traffic mutants. CPY secretion was measured in the presence and absence of 500 mm KCl or 1M sorbitol. Sorbitol served as an osmolarity control, used to test whether K^+^ specifically or increased osmolarity in general affected CPY secretion. We found that in 28 of 56 strains tested, both K^+^ and sorbitol suppressed secretion. In 10 strains, only K^+^ suppressed CPY secretion (as shown in the *vps9Δ* strain, for example). In 18 strains, neither K^+^ nor sorbitol affected CPY secretion. For the complete data set, see Table S4.

Loss of intracellular K^+^ leads to inhibition of both clathrin-mediated (Larkin *et al.*, 1986, Larkin *et al.*, 1985, Larkin *et al.*, 1983) and clathrin-independent endocytosis (Vercauteren *et al.*, 2010). We asked whether increases in extracellular K^+^ could negate the effects of loss of certain gene products involved in membrane traffic. Specifically, we examined the membrane traffic mutants from all three classes identified in the original screen by asking whether growth on medium containing increased KCl suppressed the carboxypeptidase Y (CPY) secretion phenotype exhibited by these strains. Since addition of KCl to the medium affects not only K^+^ levels but also osmolarity, we compared the CPY secretion phenotype of the strains on regular medium with no additions, with added KCl, and with added sorbitol as an osmolarity control. Of the strains tested (see Figure 6 for representative data and Table S4 for the complete data set), the CPY secretion phenotype of 10 strains was suppressed well by KCl but not by sorbitol. These are *did4Δ/vps2Δ* (class I), *pep7Δ/vps19Δ* (class II), *pep12Δ/vps6Δ* (class II), *vps1Δ* (class II), *vps3Δ* (class II), *vps4Δ* (class I), *vps8Δ* (class I), *vps9Δ* (class I), *vps24Δ* (class I), and *vps27Δ* (class I). These results are consistent with a model that some of these gene products may play structural roles in the complexes in which they function, while others are important for the catalytic function, and loss of the structural proteins can be suppressed in part by increasing the K^+^ content of cells.

### Loss of inositol kinases results in decreased steady state Trk1-HA protein levels

**Figure 7:**
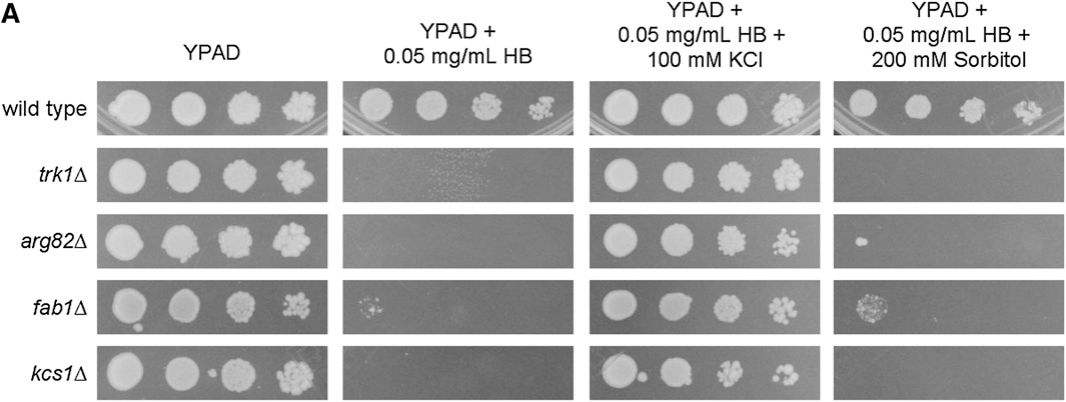

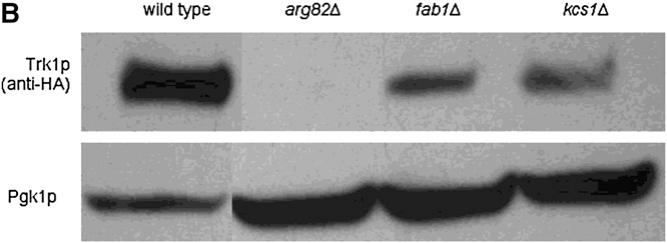
Inositol kinases are involved in the modulation of Trk1 steady state protein levels. (A) Wild-type (BY4743), *trk1∆*, *arg82∆*, *fab1∆*, and *kcs1∆* strains were grown in rich medium, then diluted to a concentration of 1 OD/mL. Ten-fold serial dilutions of the cells were spotted onto media containing HB, HB + KCl, and HB + sorbitol at the above listed concentrations, and the plates were incubated for 3 days at 30°C and then photographed. (B) Wild type, *arg82∆*, *fab1∆*, and *kcs1∆* strains were transformed with a 2 µ plasmid bearing Trk1-HA. Protein lysates were prepared from these transformed strains, and the total lysate was separated by gel electrophoresis as described in Materials and Methods and subjected to Western blotting with the anti-HA antibody. Pgk1 is shown as a loading control.

The same initial screen also identified three mutants lacking genes encoding inositol (*kcs1Δ* and *arg82Δ*) and phosphatidylinositol (*fab1Δ*) kinases, all of which have been implicated in proper vacuolar function and morphology (Cooke *et al.*, 1998, Dubois *et al.*, 2002, El Alami *et al.*, 2003, Yamamoto *et al.*, 1995, Zhang *et al.*, 2001). Loss of any of these genes resulted in hygromycin B-sensitivity that was suppressed by KCl but not equiosmolar sorbitol (Figure 7A), and all three mutants exhibited decreased Rb^+^ uptake relative to wild-type (data not shown). Transforming the plasmid containing the Trk1-HA allele into these strains and examining the expression of Trk1-HA revealed that it is expressed at lower levels in the *fab1∆* and *kcs1∆* mutants, and is not expressed at all in the *arg82∆* mutant (Figure 7B), in sharp contrast to the results from the majority of the membrane traffic mutants where for the most part, Trk1-HA levels were unaffected. This suggests that the function of these three gene products, possibly through their effects on vacuolar function, is important in the regulation of Trk1’s steady state protein levels. However, Arg82 is also involved in the regulation of arginine biosynthesis by interacting with and preventing the degradation of the transcription factors Arg80 and Mcm1 (El Alami *et al.*, 2003), and this function is separate from its involvement in vacuolar function and morphology (El Alami *et al.*, 2003, Dubois *et al.*, 2002). Therefore, further investigation is required to elucidate the functions of these proteins on Trk1 expression and activity.

### Models for the connection between regulation of membrane traffic and K^+^

Our results show that the K^+^ influx defects observed are unlinked to steady state level of localization of Trk1-HA in most strains lacking a membrane traffic regulator. In order to explain these results, we have come up with two alternative models that will form the basis of future experiments. The first is the direct model: the modulators of Trk1 activity are aberrantly trafficked as a result of loss of membrane traffic regulators. This model is consistent with the observation that in most cases, wild-type amounts of Trk1-HA are present at the plasma membrane and in the correct lipid subdomain, but nevertheless, show decreased ^86^Rb^+^ uptake. As mentioned previously, positive and negative regulators of Trk1 have been identified: the Hal kinases (Mulet *et al.*, 1999, Pérez-Valle *et al.*, 2007) and Ppz phosphatases (Posas *et al.*, 1995, Ruiz *et al.*, 2004b, Yenush *et al.*, 2005, Yenush *et al.*, 2002), respectively. If the direct model is valid, then either the positive regulators are unable to reach Trk1 or the negative regulators are unable to leave the site where Trk1 is present when membrane traffic is disrupted. This model is consistent with our observation that overexpression of Trk1 does not suppress the hygromycin B-sensitive phenotype. In further support, Ppz1 is localized to the plasma membrane and interacts with Trk1 at that site (Yenush *et al.*, 2005, Yenush *et al.*, 2002), a result confirmed by GFP tagging (Huh *et al.*, 2003). Thus, Ppz1 (and perhaps Ppz2) may be trapped at the plasma membrane in the membrane traffic mutants.

The second model, the indirect model, builds upon the observation that control of cytosolic *vs.* lumenal pH and K^+^ are important for control of membrane traffic (Brett *et al.*, 2005). In this model, Trk1 activity could be indirectly down-regulated, because the ionic conditions necessary for membrane traffic are not fulfilled. Important proteins in control of these processes include a variety of Na^+^-K^+^/H^+^ exchangers, including Kha1 (Maresova and Sychrova, 2005, Maresova and Sychrova, 2010, Ramirez *et al.*, 1998), Nha1 (Banuelos *et al.*, 2002, Banuelos *et al.*, 1998, Prior *et al.*, 1996, Sychrova *et al.*, 1999), Nhx1/Vps44 (Bowers *et al.*, 2000, Nass *et al.*, 1997, Nass and Rao 1998), and Vnx1 (Cagnac *et al.*, 2010, Cagnac *et al.*, 2007). This model predicts that loss of membrane traffic proteins responsible for the localization or activity of one or more of these Na^+^-K^+^/H^+^ exchangers results in the phenotypes observed. In support of this model with respect to Nhx1, it was previously found that a *gyp6Δ* mutant is resistant to hygromycin B, overexpression of *GYP6* confers hygromycin B sensitivity, and Gyp6 binds to Nhx1 as analyzed by two-hybrid studies (Ali *et al.*, 2004). Gyp6 thus appears to be a negative regulator of Nhx1 activity, controlling the pH of vesicles (Ali *et al.*, 2004). In addition, Gyp6 is the GTPase-activating protein for the monomeric G-protein, Ypt6 (Strom *et al.*, 1993, Will and Gallwitz 2001), while Ric1 and Rgp1 together encode the guanine–nucleotide exchange factor for Ypt6 (Siniossoglou *et al.*, 2000). In our screen, we obtained the *nhx1Δ*, *ypt6Δ*, *ric1Δ*, and *rgp1Δ* strains and all have decreased levels of ^86^Rb^+^ uptake. Interestingly, the *ypt6Δ*, *ric1Δ*, and *rgp1Δ* strains all fell into class I (sensitivity is suppressed by 100 mm KCl), but the *nhx1Δ* strain was a member of class III (its sensitivity cannot be suppressed by raising extracellular KCl). Preliminary data, however, suggest that simply overexpressing *NHX1* in the 29 class I membrane traffic mutants was not sufficient to reverse the hygromycin B-sensitive phenotype (Steidel and Rosenwald, unpublished data). We will examine localization of Nhx1 and the other exchanges in these strains in the future. Additionally, we note that these two models may not be mutually exclusive, that the means of signaling from the exchangers to Trk1 may be via the Hal and/or Ppz proteins.

## Conclusion

In this study, because several mutants with defects in K^+^ homeostasis were previously shown to be sensitive to hygromycin B, we used this phenotype as a way to identify genes with potential roles in K^+^ homeostasis. Many of the hygromycin B-sensitive mutants were defective in membrane traffic, as others have previously described, but as we show here, the vast majority of these mutants were also defective in K^+^ uptake. However, for the majority of the mutants analyzed in detail, major changes in steady state level or localization of the major K^+^ importer, Trk1, were not observed, suggesting that Trk1 activity is down-regulated in these strains, although at present whether the effect seen is as a result of our direct or indirect models remains to be determined. In addition, we demonstrated that the CPY secretion phenotype of some strains could be suppressed specifically by the addition of additional KCl in the medium but not by an equal osmolar concentration of sorbitol. Finally, we showed that loss of three inositol kinases also affected K^+^ homeostasis, but in a way that is likely dependent on the control of steady state levels of Trk1. In summary, our work highlights a significant nexus between control of intracellular K^+^ levels and regulation of membrane traffic.

## Supplementary Material

Supporting Information
