# Supplementary material for: Identification of Yeast Genes Involved in K+ Homeostasis: Loss of Membrane Traffic Genes Affects K+ Uptake
Source: G3 (Bethesda). 2011 Jun 1;1(1):43–56. doi: 10.1534/g3.111.000166 (PMC3276120; doi:10.1534/g3.111.000166)
Supplement: Supporting Information [file supp_1_1_43__index.html]

Supporting Information 

# Identification of Yeast Genes Involved in K+ Homeostasis: Loss of Membrane Traffic Genes Affects K+ Uptake

## Supporting Information for Fell *et al.*, 2011

**Files in this Data Supplement:**

- Supporting Information - Tables S1-S4 (PDF, 160 KB)
- Table S1A - Class  I  Mutants:  Hygromycin  B  Sensitive  Strains  Suppressed  by  100  mM  KCl  (PDF, 100 KB)
- Table S1B - Class II Mutants: Hygromycin B Sensitive Strains Suppressed by 500 mM KCl (PDF, 76 KB)
- Table S1C - Class  III  Mutants:  Hygromycin  B  Sensitive  Strains  Not  Suppressed  Well   by  500  mM  KCl  (PDF, 84 KB)
- Table S3 - 86Rb+ Uptake by Membrane Traffic Mutants of All Three Classes (PDF, 88 KB)
- Table S4 - Effect of K+ on CPY Secretion in Membrane Traffic Mutants (PDF, 96 KB)
- Table S2A - Alphabetical Listing of Genes with Associated GO Terms (Microsoft Excel, .xls, 128 KB)
- Table S2B - Process, Function, and Component GO Terms Arranged by Significance for the Entire Set of 156 Genes (Microsoft Excel, .xls, 132 KB)
- Table S2C - Process GO Terms Arranged by Significance for Genes in Each of the Three Classes (Microsoft Excel, .xls, 44 KB)
