# Supplementary material for: Identification of Yeast Genes Involved in K+ Homeostasis: Loss of Membrane Traffic Genes Affects K+ Uptake
Source: G3 (Bethesda). 2011 Jun 1;1(1):43–56. doi: 10.1534/g3.111.000166 (PMC3276120; doi:10.1534/g3.111.000166)
Supplement: Supporting Information [file supp_1.1.43_000166SI.pdf]

Table S1A Class I Mutants: Hygromycin B Sensitive Strains Suppressed by 100 mM KCl

Strain	ORF	Aliases	YPAD	YPAD+ 100 mM KCl	HB	HB+ 100 mM KCl
WT			**++++**	**++++**	**+++**	**++++**
Membrane Traffic Proteins (30)						
arl1Δ	YBR164C	*DLP2*	++++	++++	-	++++
bro1Δ	YPL084W	*VPS31*	++++	++++	-	++++
chs5Δ	YLR330W	*CAL3*	++++	++++	-	++++
cog5Δ	YNL051W	*COD4*	++++	++++	+/-	++++
cog6Δ	YNL041C	*COD2*	++++	++++	+	++++
did4Δ	YKL002W	*VPS2*	++++	++++	-	++++
gga1Δ	YDR358W		++++	++++	+	++++
gga2Δ	YHR108W		++++	++++	-	++++
glo3Δ	YER122C		+++	+++	-	+++
gos1Δ	YHL031C		++++	++++	+/-	+++
mon2Δ	YNL297C	*YSL2*	++++	++++	+/-	+++
pep5D	YMR231W	*VAM1, VPS11*	++++	++++	-	++++
rer1Δ	YCL001W		++++	++++	-	+++
rgp1Δ	YDR137W		++++	++++	+/-	++++
ric1Δ	YLR039C		++++	++++	+	++++
sec22Δ	YLR268W	*SLY2, TSL26*	++++	++++	-	+++
stp22D	YCL008C	*VPS23*	++++	++++	-	++++
vam3Δ	YOR106W	*PTH1*	++++	++++	-	++++
vam7Δ	YGL212W	*VPS43*	++++	++++	-	++++
vps4Δ	YPR173C	*DID6, GRD13*	++++	++++	-	++++
vps8Δ	YAL002W	*FUN15, VPT8*	++++	++++	-	++++
vps9Δ	YML097C	*VPL31, VPT9*	++++	++++	-	++++
vps20Δ	YMR077C		++++	++++	-	++++
vps21Δ	YOR089C	*YPT51*	+++	++++	-	++++
vps24Δ	YKL041W	*DID3*	++++	++++	-	++++
vps27Δ	YNR006W	*GRD11, DID7*	++++	++++	-	++++
vps30Δ	YPL120W	*APG6, VPT30*	++++	++++	-	++++
vps36Δ	YLR417W	*GRD12, VAC3*	++++	++++	-	++++
vps41Δ	YDR080W	*VAM2, VPL20*	++++	++++	-	+++
ypt6Δ	YLR262C		++++	++++	+/-	++++
Ion Transporters (2)						
gef1Δ	YJR040W	*CLC*	++++	++++	-	+++

trk1Δ	YJL129C		++++	++++	-	++++
Protein Kinases (2)						
hal5Δ	YJL165C		++++	++++	-	++++
sat4D	YCR008W	*HAL4*	++++	++++	-	++++
Glycosylation (3)						
alg6Δ	YOR002W		++++	++++	-	++++
hoc1Δ	YJR075W		++++	++++	+	++++
van1Δ	YML115C		++++	++++	+	++++
Inositol kinases (3)						
arg82Δ	YDR173C	*IPK2*	++++	++++	-	++
fab1Δ	YFR019W	*SVL7*	+++	+++	+	+++
kcs1Δ	YDR017C		++++	++++	-	+++
Metabolism (2)						
adh1Δ	YOL086C		++++	++++	-	++++
ure2Δ	YNL229C		++++	++++	-	++++
Miscellaneous (16)						
arv1Δ	YLR242C		++++	++++	-	+++
bem1Δ	YBR200W		++++	++++	-	+++
cdc50Δ	YCR094W		++++	++++	-	+++
cyt1Δ	YOR065W	*CTC1*	+++	+++	+/-	+++
eft2Δ	YDR385W		++++	++++	-	+++
kap120Δ	YPL125W		++++	++++	-	++++
lsb3D	YFR024C		++++	++++	-	++++
nat3Δ	YPR131C		++++	++++	-	+++
nbp2Δ	YDR162C		++++	++++	-	++++
ncs6D	YGL211W		++++	++++	-	++++
ram1Δ	YDL090C		++++	++++	+	++++
reg1Δ	YDR028C		++++	++++	+/-	+++
sap155Δ	YFR040W		++++	++++	+/-	++++
sse1Δ	YPL106C		++++	++++	-	+++
vph2Δ	YKL119C	*CLS10, VMA12*	++++	++++	-	++++
Transcription / Replication (12)						
csi2Δ	YOL007C		++++	++++	-	+++
ctf4Δ	YPR135W		+++	+++	-	+++
eaf1Δ	YDR359C	*VID21*	++++	++++	-	+++
irs4Δ	YKR019C		++++	++++	+	++++
mdm20Δ	YOL076W		++++	++++	+	++++

rad6Δ	YGL058W		++++	++++	-	++++
rtg1Δ	YOL067C		+++	+++	+/-	++++
scp160Δ	YJL080C		++++	++++	+	++++
sin3Δ	YOL004W		++++	++++	+	++++
sto1Δ	YMR125W		++++	++++	-	++++
tho2Δ	YNL139C	*RLR1*	+++	+++	-	+++
tup1Δ	YCR084C		+++	+++	-	+++
Ribosomal Proteins (4)						
rpl21aΔ	YBR191W		++++	++++	+/-	++++
rpl22aΔ	YLR061W		++++	++++	-	+++
rpl27aΔ	YHR010W		++++	++++	-	+++
rpp1bΔ	YDL130W		++++	++++	+/-	++++
Unknown Function (3)						
fyv4Δ	YHR059W		++++	++++	-	+++
smi1Δ	YGR229C		++++	++++	-	++++
	YDL133W		++++	++++	+	++++

Strains from the deletion collection (Winzeler *et al.*, 1999) were screened for growth on medium containing 0.1 mg/ml hygromycin B compared to growth on medium without drug. The 156 strains listed in Tables S1A-S1C were sensitive to hygromycin B (HB). The set of strains was further separated into three classes by the ability of KCl to suppress hygromycin B sensitive growth (0.075 – 0.1 mg/ml). The strains shown here were able to grow in the presence of hygromycin B if the medium was supplemented with 100 mM KCl. The membrane traffic mutants here were studied in more detail. See Table 3 and Figures 3 – 6 for details

Table S1B   Class II Mutants: Hygromycin B Sensitive Strains Suppressed by 500 mM KCl

Strain	ORF	Aliases	YPAD	YPAD+500 mM KCl	HB	HB+500 mM KCl
WT			++++	++++	+++	++++
Membrane Traffic Proteins (12)						
arf1Δ	YDL192W		++++	++++	-	+++
mon1Δ	YGL124C		++++	++++	++	++++
pep7Δ	YDR323C	*VPS19, VAC1*	++++	++++	-	++++
pep12Δ	YOR036W	*VPS6, VPT13*	++++	++++	-	+++
per1Δ	YCR044C	*COS16*	++++	++++	-	++
swa2Δ	YDR320C	*AUX1, BUD24*	++++	++++	-	+++
sys1Δ	YJL004C		++++	++++	++	++++
vps1Δ	YKR001C	*GRD1, VPT26*	++++	++++	++	+++
vps3Δ	YDR495C	*PEP6, VPT17*	++++	++++	-	+++
vps29Δ	YHR012W	*PEP11*	++++	++++	-	++++
vps52Δ	YDR484W	*SAC2*	++++	++++	-	+++
vps75D	YNL246W		++++	+++	++	++++
Miscellaneous (7)						
grr1Δ	YJR090C	*CAT80, COT2*	++++	++++	+	+++
hal3Δ	YKR072C	*SIS2*	++++	++++	++	++++
met22Δ	YOL064C	*HAL2*	++++	++++	++	++++
ncs2D	YNL119W		++++	++++	++	++++
slm4D	YBR077C		++++	++++	++	++++
snf3Δ	YDL194W		++++	++++	++	++++
ubx4D	YMR067C		++++	++++	++	++++
Transcription and Replication (2)						
ist3Δ	YIR005W	*SNU17*	++++	++++	++	++++
xrs2Δ	YDR369C		+++	+++	+	+++
Unknown (2)						
ilm1Δ	YJR118C		++++	++++	++	++++
	YDR161W		++++	++++	++	+++

As in the legend for Table S1A, strains were grown +/- 0.075 mg/ml hygromycin B but +/- 500 mM KCl. Strains that grew as well in the presence of hygromycin B and 500 mM KCl as they did without either addition were denoted Class II. Several strains grew modestly in the presence of hygromycin B if 100 mM KCl was added, but 500 mM was needed to achieve the same level of growth as in the absence of additions. Twenty-three strains fell into Class II.

Table S1C Class III Mutants: Hygromycin B Sensitive Strains Not Suppressed Well by 500 mM KCl

Gene	ORF	Aliases	YPAD	YPAD+500 mM KCl	HB	HB+500 mM KCl
WT			++++	++++	+++	++++
Membrane Traffic Proteins (14)						
apl2Δ	YKL135C		++++	++++	-	+
get1Δ	YGL020C	*MDM39*	++++	++++	-	++
get2Δ	YER083C	*RMD7*	++++	++++	-	+
nhx1Δ	YDR456W	*VPS44*	++++	++++	-	+/-
pep3Δ	YLR148W	*VPS18, VAM8*	++++	++++	-	-
rvs161Δ	YCR009C	*END6, FUS7*	++++	++++	-	+/-
rvs167Δ	YDR388W		++++	++++	-	-
snx3D	YOR357C	*GRD19*	+++	++++	-	+
tlg2Δ	YOL018C		++++	++++	-	+/-
vps16Δ	YPL045W	*VAM9, VPT16*	++++	++++	-	-
vps33Δ	YLR396C	*PEP14, VAM5*	++++	++++	-	-
vps51Δ	YKR020W	*WHI6, API3*	++++	++++	-	++
vps54Δ	YDR027C	*LUV1*	+++	+++	-	-
vps53Δ	YJL029C		++++	++++	-	-
Phosphatases (3)						
ptc1Δ	YDL006W	*KCS2, TPD1*	++++	++++	-	++
sac1Δ	YKL212W	*RSD1*	++++	++++	-	-
sit4Δ	YDL047W	*LGN4*	+++	+++	+	++
Glycosylation (3)						
anp1Δ	YEL036C	*MNN8, GEM3*	+++	+++	-	++
gup1Δ	YGL084C		++++	++++	-	++
rot2Δ	YBR229C	*GLS2*	++++	++++	-	+
Lipid Metabolism (3)						
erg3Δ	YLR056W	*SYR1, PSO6*	++++	++++	-	+
erg28Δ	YER044C	*BUD18*	++++	++++	-	-
plc1Δ	YPL268W		+++	+++	-	+
Miscellaneous (9)						
adk1Δ	YDR226W	*AKY1*	+++	+++	-	+/-
bur2Δ	YLR226W	*CST4*	+++	+++	-	-
gas1Δ	YMR307W	*GGP1, CWH52*	+++	++++	-	+
gtr2Δ	YGR163W		++++	++++	+	++
nup133Δ	YKR082W		+++	+++	+/-	++

pho80Δ	YOL001W	*VAC5, TUP7*	++++	++++	-	++
pmp3Δ	YDR276C		++++	++++	-	-
shp1Δ	YBL058W		++++	++++	-	++
slg1Δ	YOR008C	*HCS77, WSC1*	++++	++++	-	++
Transcription and Replication (22)						
bdf1Δ	YLR399C		+++	+++	-	+
cdc40Δ	YDR364C	*PRP17, SLT15*	+++	++++	-	-
ctk3Δ	YML112W		+++	+++	-	+
dbp7Δ	YKR024C		+++	+++	+/-	++
dhh1Δ	YDL160C		+++	+++	-	++
hap5Δ	YOR358W		++++	++++	-	+
hmo1Δ	YDR147W	*HSM2*	+++	+++	-	++
imp2Δ	YIL154C		++++	++++	-	+
not5Δ	YPR072W		+++	+++	-	+
pol32Δ	YJR043C		++++	++++	++	+++
rad50Δ	YNL250W		+++	+++	+	++
ref2Δ	YDR195W		++	++	-	+
rox3Δ	YBL093C	*NUT3, SSN7*	++++	++++	-	+/-
rpb9Δ	YGL070C	*SHI1, SSU73*	++++	++++	-	+
sfp1Δ	YLR403W		+++	+++	-	-
spt20Δ	YOL148C	*ADA5*	+++	+++	-	+/-
srb2Δ	YHR041C	*HRS2*	++++	++++	-	++
srb5Δ	YGR104C		+++	+++	-	+
ssz1Δ	YHR064C	*PDR13*	++++	++++	-	-
taf14Δ	YPL129W	*ANC1, SWP29*	++++	++++	-	+
tif4631Δ	YGR162W		++++	++++	-	++
zuo1Δ	YGR285C		++++	++++	-	-
Ribosomal Proteins (1)						
rpl31aΔ	YDL075W		+/-	+/-	-	-
Unknown Function (2)						
	YDR532C		++	+++	-	+/-
	YOL015W		++++	++++	-	++

As in the legend for Table S1B, strains were growth +/- 0.075 mg/ml hygromycin B and +/- 500 mM KCl. Strains unable to grow to the same extent in the presence of hygromycin B and 500 mM KCl as they do in the absence of the two additions were denoted as members of Class III. Fifty-seven strains fell into this category.

Table S2 Gene Ontology (GO) Terms

Tables S2A-S2C are available for download at http://www.g3journal.org/lookup/suppl/doi:10.1534/g3.111.000166/-/DC1.

Tables S2A: Alphabetical Listing of Genes with Associated GO Terms

Tables S2B: Process, Function, and Component GO Terms Arranged by Significance for the Entire Set of 156 Genes

Tables S2C: Process GO Terms Arranged by Significance for Genes in Each of the Three Classes

Note that each file has multiple tabs at bottom.

Table S3 $^{86}Rb^+$ Uptake by Membrane Traffic Mutants of All Three Classes

Class	Strain	ORF	Percent $^{86}Rb^+$ Uptake
	WT		100%
**1**	***arl1Δ***	**YBR164C**	**68%**
**1**	***bro1Δ***	**YPL084W**	**8%**
**1**	***chs5Δ***	**YLR330W**	**33%**
**1**	***cog5Δ***	**YNL051W**	**67%**
1	*cog6Δ*	YNL041C	116%
**1**	***did4Δ***	**YKL002W**	**62%**
1	*gga1Δ*	YDR358W	81%
1	*gga2Δ*	YHR108W	122%
**1**	***glo3Δ***	**YER122C**	**41%**
**1**	***gos1Δ***	**YHL031C**	**38%**
**1**	***mon2Δ***	**YNL297C**	**34%**
1	*pep5D*	YMR231W	76%
1	*rer1Δ*	YCL001W	151%
**1**	***rgp1Δ***	**YDR137W**	**56%**
**1**	***ric1Δ***	**YLR039C**	**47%**
**1**	***sec22Δ***	**YLR268W**	**49%**
**1**	***stp22Δ***	**YCL008C**	**13%**
**1**	***vam3Δ***	**YOR106W**	**75%**
**1**	***vam7Δ***	**YGL212W**	**39%**
**1**	***vps4Δ***	**YPR173C**	**55%**
**1**	***vps8Δ***	**YAL002W**	**58%**
**1**	***vps9Δ***	**YML097C**	**61%**
**1**	***vps20Δ***	**YMR077C**	**75%**
**1**	***vps21Δ***	**YOR089C**	**56%**
**1**	***vps24Δ***	**YKL041W**	**23%**
1	*vps27Δ*	YNR006W	90%
**1**	***vps30Δ***	**YPL120W**	**47%**
**1**	***vps36Δ***	**YLR417W**	**29%**
**1**	***vps41Δ***	**YDR080W**	**52%**
**1**	***ypt6Δ***	**YLR262C**	**54%**

**2**	***arf1Δ***	**YDL192W**	**58%**
**2**	***mon1Δ***	**YGL124C**	**49%**
**2**	***pep7Δ***	**YDR323C**	**38%**
**2**	***pep12Δ***	**YOR036W**	**48%**
**2**	***per1Δ***	**YCR044C**	**52%**
**2**	***swa2Δ***	**YDR320C**	**0%**
**2**	***sys1Δ***	**YJL004C**	**66%**
**2**	***vps1Δ***	**YKR001C**	**24%**
**2**	***vps3Δ***	**YDR495C**	**33%**
**2**	***vps29Δ***	**YHR012W**	**47%**
2	*vps52Δ*	YDR484W	109%
**2**	***vps75Δ***	**YNL246W**	**21%**
**3**	***apl2Δ***	**YKL135C**	**64%**
3	*get1D*	YGL020C	n.d.
3	*get2D*	YER083C	n.d.
**3**	***nhx1Δ***	**YDR456W**	**20%**
**3**	***pep3Δ***	**YLR148W**	**46%**
3	*rvs161D*	YCR009C	n.d.
3	*rvs167D*	YDR388W	n.d.
**3**	***snx3Δ***	**YOR357C**	**10%**
3	*tlg2Δ*	YOL018C	<u>276%</u>
**3**	***vps16Δ***	**YPL045W**	**45%**
**3**	***vps33Δ***	**YLR396C**	**47%**
3	*vps51Δ*	YKR020W	<u>372%</u>
3	*vps53Δ*	YJL029C	<u>142%</u>
3	*vps54Δ*	YDR027C	106%

Cells were incubated with ^{86}RbCl as described in the legend to Figure 2. Aliquots were removed at time 0 and at 30 min. Uptake was determined in triplicate. Each strain was tested at least twice on different days; the average of the independent determinations relative to wild type is shown. Results between experiments generally varied by less than 10%. The different mutants were sorted into 3 separate bins: strains exhibiting <75% of wild type uptake were conserved to be defective for uptake (in **bold**). Strains exhibiting >125% of wild type were considered to have excess uptake (<u>underlined</u>) and were examined for $^{86}Rb^{+}$ efflux as described in the text. Strains exhibiting between >75% but <125% of wild type were considered to be indistinguishable from wild type.

Table S4 Effect of K^+ on CPY Secretion in Membrane Traffic Mutants

Class	Gene	ORF	CPY	CPY on KCl	CPY on Sorb
-	WT	-	-	-	-
1	*arl1Δ*	YBR164C	+++	+/-	+/-
1	*bro1Δ*	YPL084W	++++	-	-
1	*chs5D*	YLR330W		*non-secretor*	
1	*cog5Δ*	YNL051W	++++	+	-
1	*cog6Δ*	YNL041C	++++	+	-
**1**	***did4Δ***	**YKL002W**	**+++**	**-**	**++**
1	*gef1D*	YJR040W	++	-	-
1	*gga1Δ*	YDR358W	+++	-	-
1	*gga2Δ*	YHR108W	+++	-	-
1	*glo3D*	YER122C	+/-	-	-
1	*gos1Δ*	YHL031C	++++	++	+
1	*mdm20D*	YOL076W	+/-	-	-
1	*mon2D*	YNL297C	+/-	+/-	+/-
1	*pep5Δ*	YMR231W	++++	++++	++++
1	*rer1D*	YCL001W		*non-secretor*	
1	*rgp1Δ*	YDR137W	++++	+	+
1	*ric1Δ*	YLR039C	++++	+++	+++
1	*sec22Δ*	YLR268W	+++	+	+
1	*stp22Δ*	YCL008C	++++	-	-
1	*vam3D*	YOR106W	+/-	-	-
1	*vam7Δ*	YGL212W	++++	+++	+++
1	*van1D*	YML115C	++	-	-
1	*vph2Δ*	YKL119C	+++	-	-
**1**	***vps4Δ***	**YPR173C**	**++++**	**+/-**	**+++**
**1**	***vps8Δ***	**YAL002W**	**++++**	**+/-**	**+++**
**1**	***vps9Δ***	**YML097C**	**++++**	**+/-**	**+++**
1	*vps20Δ*	YMR077C	++++	+/-	+/-
1	*vps21Δ*	YOR089C	++++	+	+
**1**	***vps24Δ***	**YKL041W**	**++++**	**+/-**	**+++**
**1**	***vps27Δ***	**YNR006W**	**++++**	**+/-**	**+++**
1	*vps30Δ*	YPL120W	++++	++++	++++
1	*vps36Δ*	YLR417W	++++	+	+
1	*vps41Δ*	YDR080W	++++	++	++
1	*ypt6Δ*	YLR262C	++++	+++	++++

2	*arf1Δ*	YDL192W	++++	+++	+++
2	*mon1D*	YGL124C	+++	+++	++
**2**	***pep7Δ***	**YDR323C**	**++++**	**-**	**++**
**2**	***pep12Δ***	**YOR036W**	**++++**	**-**	**++**
2	*per1Δ*	YCR044C	+++	-	-
2	*swa2Δ*	YDR320C	+++	+	+
2	*sys1Δ*	YJL004C	++++	+	+
**2**	***vps1Δ***	**YKR001C**	**++++**	**+**	**+++**
**2**	***vps3Δ***	**YDR495C**	**++++**	**-**	**+++**
2	*vps29Δ*	YHR012W	++++	++++	++++
2	*vps52Δ*	YDR484W	++++	+++	++++
2	*vps75Δ*	YNL246W	++++	+/-	+/-
3	*nhx1Δ*	YDR456W	++++	++++	++++
3	*pep3Δ*	YLR148W	++++	+++	+++
3	*ptc1D*	YDL006W	++	-	-
3	*snx3D*	YOR357C	++	-	-
3	*tlg2Δ*	YOL018C	++++	++++	++++
3	*vps16Δ*	YPL045W	++++	++++	+++
3	*vps33Δ*	YLR396C	++++	++	++
3	*vps51Δ*	YKR020W	++++	+++	+++
3	*vps53Δ*	YJL029C	++++	++++	++++
3	*vps54Δ*	YDR027C	++++	+++	+++

The membrane traffic mutants in Classes I, II, and III were compared to the strains known to secrete CPY (Bonangelino *et al.*, 2002). We included in our analysis 5 strains obtained in our screen which secrete CPY but do not have GO terms that connote membrane traffic (*gef1D*, *mdm20D*, *van1D*, *vph2D* and *ptc1D;* see Tables S2A). All were grown overnight in rich medium, diluted to 1.0 OD_{600}/ml, then subjected to serial 10-fold dilutions. Cells were spotted onto rich medium without or with the additions of 0.5 M KCl or 1 M sorbitol using a replicator tool and grown overnight at 30°C. The next day, cells were overlaid with a nitrocellulose filter. After 15 -18 h, the filter was removed, washed free of cells, and prepared for Western analysis using a monoclonal anti-CPY antibody (Roberts *et al.*, 1991). Strains are listed by class as in Tables S1A-C and strains in which KCl specifically suppressed secretion are highlighted in bold. - = no secretion, +/- = minimal secretion, + or ++ = moderate secretion, and +++ or ++++ = large amounts of secreted CPY.
