# Supplementary material for: Identification of Yeast Genes Involved in K+ Homeostasis: Loss of Membrane Traffic Genes Affects K+ Uptake
Source: G3 (Bethesda). 2011 Jun 1;1(1):43–56. doi: 10.1534/g3.111.000166 (PMC3276120; doi:10.1534/g3.111.000166)
Supplement: Supporting Information [file supp_1.1.43_TableS1A.pdf]

Table S1A Class I Mutants: Hygromycin B Sensitive Strains Suppressed by 100 mM KCl

Strain	ORF	Aliases	YPAD	YPAD+ 100 mM KCl	HB	HB+ 100 mM KCl
WT			**++++**	**++++**	**+++**	**++++**
Membrane Traffic Proteins (30)						
arl1Δ	YBR164C	*DLP2*	++++	++++	-	++++
bro1Δ	YPL084W	*VPS31*	++++	++++	-	++++
chs5Δ	YLR330W	*CAL3*	++++	++++	-	++++
cog5Δ	YNL051W	*COD4*	++++	++++	+/-	++++
cog6Δ	YNL041C	*COD2*	++++	++++	+	++++
did4Δ	YKL002W	*VPS2*	++++	++++	-	++++
gga1Δ	YDR358W		++++	++++	+	++++
gga2Δ	YHR108W		++++	++++	-	++++
glo3Δ	YER122C		+++	+++	-	+++
gos1Δ	YHL031C		++++	++++	+/-	+++
mon2Δ	YNL297C	*YSL2*	++++	++++	+/-	+++
pep5D	YMR231W	*VAM1, VPS11*	++++	++++	-	++++
rer1Δ	YCL001W		++++	++++	-	+++
rgp1Δ	YDR137W		++++	++++	+/-	++++
ric1Δ	YLR039C		++++	++++	+	++++
sec22Δ	YLR268W	*SLY2, TSL26*	++++	++++	-	+++
stp22D	YCL008C	*VPS23*	++++	++++	-	++++
vam3Δ	YOR106W	*PTH1*	++++	++++	-	++++
vam7Δ	YGL212W	*VPS43*	++++	++++	-	++++
vps4Δ	YPR173C	*DID6, GRD13*	++++	++++	-	++++
vps8Δ	YAL002W	*FUN15, VPT8*	++++	++++	-	++++
vps9Δ	YML097C	*VPL31, VPT9*	++++	++++	-	++++
vps20Δ	YMR077C		++++	++++	-	++++
vps21Δ	YOR089C	*YPT51*	+++	++++	-	++++
vps24Δ	YKL041W	*DID3*	++++	++++	-	++++
vps27Δ	YNR006W	*GRD11, DID7*	++++	++++	-	++++
vps30Δ	YPL120W	*APG6, VPT30*	++++	++++	-	++++
vps36Δ	YLR417W	*GRD12, VAC3*	++++	++++	-	++++
vps41Δ	YDR080W	*VAM2, VPL20*	++++	++++	-	+++
ypt6Δ	YLR262C		++++	++++	+/-	++++
Ion Transporters (2)						
gef1Δ	YJR040W	*CLC*	++++	++++	-	+++

trk1Δ	YJL129C		++++	++++	-	++++
Protein Kinases (2)						
hal5Δ	YJL165C		++++	++++	-	++++
sat4D	YCR008W	*HAL4*	++++	++++	-	++++
Glycosylation (3)						
alg6Δ	YOR002W		++++	++++	-	++++
hoc1Δ	YJR075W		++++	++++	+	++++
van1Δ	YML115C		++++	++++	+	++++
Inositol kinases (3)						
arg82Δ	YDR173C	*IPK2*	++++	++++	-	++
fab1Δ	YFR019W	*SVL7*	+++	+++	+	+++
kcs1Δ	YDR017C		++++	++++	-	+++
Metabolism (2)						
adh1Δ	YOL086C		++++	++++	-	++++
ure2Δ	YNL229C		++++	++++	-	++++
Miscellaneous (16)						
arv1Δ	YLR242C		++++	++++	-	+++
bem1Δ	YBR200W		++++	++++	-	+++
cdc50Δ	YCR094W		++++	++++	-	+++
cyt1Δ	YOR065W	*CTC1*	+++	+++	+/-	+++
eft2Δ	YDR385W		++++	++++	-	+++
kap120Δ	YPL125W		++++	++++	-	++++
lsb3D	YFR024C		++++	++++	-	++++
nat3Δ	YPR131C		++++	++++	-	+++
nbp2Δ	YDR162C		++++	++++	-	++++
ncs6D	YGL211W		++++	++++	-	++++
ram1Δ	YDL090C		++++	++++	+	++++
reg1Δ	YDR028C		++++	++++	+/-	+++
sap155Δ	YFR040W		++++	++++	+/-	++++
sse1Δ	YPL106C		++++	++++	-	+++
vph2Δ	YKL119C	*CLS10, VMA12*	++++	++++	-	++++
Transcription / Replication (12)						
csi2Δ	YOL007C		++++	++++	-	+++
ctf4Δ	YPR135W		+++	+++	-	+++
eaf1Δ	YDR359C	*VID21*	++++	++++	-	+++
irs4Δ	YKR019C		++++	++++	+	++++
mdm20Δ	YOL076W		++++	++++	+	++++

rad6Δ	YGL058W		++++	++++	-	++++
rtg1Δ	YOL067C		+++	+++	+/-	++++
scp160Δ	YJL080C		++++	++++	+	++++
sin3Δ	YOL004W		++++	++++	+	++++
sto1Δ	YMR125W		++++	++++	-	++++
tho2Δ	YNL139C	*RLR1*	+++	+++	-	+++
tup1Δ	YCR084C		+++	+++	-	+++
Ribosomal Proteins (4)						
rpl21aΔ	YBR191W		++++	++++	+/-	++++
rpl22aΔ	YLR061W		++++	++++	-	+++
rpl27aΔ	YHR010W		++++	++++	-	+++
rpp1bΔ	YDL130W		++++	++++	+/-	++++
Unknown Function (3)						
fyv4Δ	YHR059W		++++	++++	-	+++
smi1Δ	YGR229C		++++	++++	-	++++
	YDL133W		++++	++++	+	++++

Strains from the deletion collection (Winzeler *et al.*, 1999) were screened for growth on medium containing 0.1 mg/ml hygromycin B compared to growth on medium without drug. The 156 strains listed in Tables S1A-S1C were sensitive to hygromycin B (HB). The set of strains was further separated into three classes by the ability of KCl to suppress hygromycin B sensitive growth (0.075 – 0.1 mg/ml). The strains shown here were able to grow in the presence of hygromycin B if the medium was supplemented with 100 mM KCl. The membrane traffic mutants here were studied in more detail. See Table 3 and Figures 3 – 6 for details
