# Supplementary material for: Identification of Yeast Genes Involved in K+ Homeostasis: Loss of Membrane Traffic Genes Affects K+ Uptake
Source: G3 (Bethesda). 2011 Jun 1;1(1):43–56. doi: 10.1534/g3.111.000166 (PMC3276120; doi:10.1534/g3.111.000166)
Supplement: Supporting Information [file supp_1.1.43_TableS1B.pdf]

Table S1B Class II Mutants: Hygromycin B Sensitive Strains Suppressed by 500 mM KCl

Strain	ORF	Aliases	YPAD	YPAD+500 mM KCl	HB	HB+500 mM KCl
WT			++++	++++	+++	++++
Membrane Traffic Proteins (12)						
arf1Δ	YDL192W		++++	++++	-	+++
mon1Δ	YGL124C		++++	++++	++	++++
pep7Δ	YDR323C	*VPS19, VAC1*	++++	++++	-	++++
pep12Δ	YOR036W	*VPS6, VPT13*	++++	++++	-	+++
per1Δ	YCR044C	*COS16*	++++	++++	-	++
swa2Δ	YDR320C	*AUX1, BUD24*	++++	++++	-	+++
sys1Δ	YJL004C		++++	++++	++	++++
vps1Δ	YKR001C	*GRD1, VPT26*	++++	++++	++	+++
vps3Δ	YDR495C	*PEP6, VPT17*	++++	++++	-	+++
vps29Δ	YHR012W	*PEP11*	++++	++++	-	++++
vps52Δ	YDR484W	*SAC2*	++++	++++	-	+++
vps75D	YNL246W		++++	+++	++	++++
Miscellaneous (7)						
grr1Δ	YJR090C	*CAT80, COT2*	++++	++++	+	+++
hal3Δ	YKR072C	*SIS2*	++++	++++	++	++++
met22Δ	YOL064C	*HAL2*	++++	++++	++	++++
ncs2D	YNL119W		++++	++++	++	++++
slm4D	YBR077C		++++	++++	++	++++
snf3Δ	YDL194W		++++	++++	++	++++
ubx4D	YMR067C		++++	++++	++	++++
Transcription and Replication (2)						
ist3Δ	YIR005W	*SNU17*	++++	++++	++	++++
xrs2Δ	YDR369C		+++	+++	+	+++
Unknown (2)						
ilm1Δ	YJR118C		++++	++++	++	++++
	YDR161W		++++	++++	++	+++

As in the legend for Table S1A, strains were grown +/- 0.075 mg/ml hygromycin B but +/- 500 mM KCl. Strains that grew as well in the presence of hygromycin B and 500 mM KCl as they did without either addition were denoted Class II. Several strains grew modestly in the presence of hygromycin B if 100 mM KCl was added, but 500 mM was needed to achieve the same level of growth as in the absence of additions. Twenty-three strains fell into Class II.
