# Supplementary material for: Identification of Yeast Genes Involved in K+ Homeostasis: Loss of Membrane Traffic Genes Affects K+ Uptake
Source: G3 (Bethesda). 2011 Jun 1;1(1):43–56. doi: 10.1534/g3.111.000166 (PMC3276120; doi:10.1534/g3.111.000166)
Supplement: Supporting Information [file supp_1.1.43_TableS1C.pdf]

Table S1C   Class III Mutants: Hygromycin B Sensitive Strains Not Suppressed Well by 500 mM KCl

Gene	ORF	Aliases	YPAD	YPAD+500 mM KCl	HB	HB+500 mM KCl
WT			++++	++++	+++	++++
Membrane Traffic Proteins (14)						
apl2Δ	YKL135C		++++	++++	-	+
get1Δ	YGL020C	*MDM39*	++++	++++	-	++
get2Δ	YER083C	*RMD7*	++++	++++	-	+
nhx1Δ	YDR456W	*VPS44*	++++	++++	-	+/-
pep3Δ	YLR148W	*VPS18, VAM8*	++++	++++	-	-
rvs161Δ	YCR009C	*END6, FUS7*	++++	++++	-	+/-
rvs167Δ	YDR388W		++++	++++	-	-
snx3D	YOR357C	*GRD19*	+++	++++	-	+
tlg2Δ	YOL018C		++++	++++	-	+/-
vps16Δ	YPL045W	*VAM9, VPT16*	++++	++++	-	-
vps33Δ	YLR396C	*PEP14, VAM5*	++++	++++	-	-
vps51Δ	YKR020W	*WHI6, API3*	++++	++++	-	++
vps54Δ	YDR027C	*LUV1*	+++	+++	-	-
vps53Δ	YJL029C		++++	++++	-	-
Phosphatases (3)						
ptc1Δ	YDL006W	*KCS2, TPD1*	++++	++++	-	++
sac1Δ	YKL212W	*RSD1*	++++	++++	-	-
sit4Δ	YDL047W	*LGN4*	+++	+++	+	++
Glycosylation (3)						
anp1Δ	YEL036C	*MNN8, GEM3*	+++	+++	-	++
gup1Δ	YGL084C		++++	++++	-	++
rot2Δ	YBR229C	*GLS2*	++++	++++	-	+
Lipid Metabolism (3)						
erg3Δ	YLR056W	*SYR1, PSO6*	++++	++++	-	+
erg28Δ	YER044C	*BUD18*	++++	++++	-	-
plc1Δ	YPL268W		+++	+++	-	+
Miscellaneous (9)						
adk1Δ	YDR226W	*AKY1*	+++	+++	-	+/-
bur2Δ	YLR226W	*CST4*	+++	+++	-	-
gas1Δ	YMR307W	*GGP1, CWH52*	+++	++++	-	+
gtr2Δ	YGR163W		++++	++++	+	++
nup133Δ	YKR082W		+++	+++	+/-	++

pho80Δ	YOL001W	*VAC5, TUP7*	++++	++++	-	++
pmp3Δ	YDR276C		++++	++++	-	-
shp1Δ	YBL058W		++++	++++	-	++
slg1Δ	YOR008C	*HCS77, WSC1*	++++	++++	-	++
Transcription and Replication (22)						
bdf1Δ	YLR399C		+++	+++	-	+
cdc40Δ	YDR364C	*PRP17, SLT15*	+++	++++	-	-
ctk3Δ	YML112W		+++	+++	-	+
dbp7Δ	YKR024C		+++	+++	+/-	++
dhh1Δ	YDL160C		+++	+++	-	++
hap5Δ	YOR358W		++++	++++	-	+
hmo1Δ	YDR147W	*HSM2*	+++	+++	-	++
imp2Δ	YIL154C		++++	++++	-	+
not5Δ	YPR072W		+++	+++	-	+
pol32Δ	YJR043C		++++	++++	++	+++
rad50Δ	YNL250W		+++	+++	+	++
ref2Δ	YDR195W		++	++	-	+
rox3Δ	YBL093C	*NUT3, SSN7*	++++	++++	-	+/-
rpb9Δ	YGL070C	*SHI1, SSU73*	++++	++++	-	+
sfp1Δ	YLR403W		+++	+++	-	-
spt20Δ	YOL148C	*ADA5*	+++	+++	-	+/-
srb2Δ	YHR041C	*HRS2*	++++	++++	-	++
srb5Δ	YGR104C		+++	+++	-	+
ssz1Δ	YHR064C	*PDR13*	++++	++++	-	-
taf14Δ	YPL129W	*ANC1, SWP29*	++++	++++	-	+
tif4631Δ	YGR162W		++++	++++	-	++
zuo1Δ	YGR285C		++++	++++	-	-
Ribosomal Proteins (1)						
rpl31aΔ	YDL075W		+/-	+/-	-	-
Unknown Function (2)						
	YDR532C		++	+++	-	+/-
	YOL015W		++++	++++	-	++

As in the legend for Table S1B, strains were growth +/- 0.075 mg/ml hygromycin B and +/- 500 mM KCl. Strains unable to grow to the same extent in the presence of hygromycin B and 500 mM KCl as they do in the absence of the two additions were denoted as members of Class III. Fifty-seven strains fell into this category.
