# Supplementary material for: Identification of Yeast Genes Involved in K+ Homeostasis: Loss of Membrane Traffic Genes Affects K+ Uptake
Source: G3 (Bethesda). 2011 Jun 1;1(1):43–56. doi: 10.1534/g3.111.000166 (PMC3276120; doi:10.1534/g3.111.000166)
Supplement: Supporting Information [file supp_1.1.43_TableS3.pdf]

**Table S3 $^{86}Rb^+$ Uptake by Membrane Traffic Mutants of All Three Classes**

| Class | Strain | ORF | Percent $^{86}Rb^+$ Uptake |
|---|---|---|---|
| | WT | | 100% |
| **1** | ***arl1Δ*** | **YBR164C** | **68%** |
| **1** | ***bro1Δ*** | **YPL084W** | **8%** |
| **1** | ***chs5Δ*** | **YLR330W** | **33%** |
| **1** | ***cog5Δ*** | **YNL051W** | **67%** |
| 1 | *cog6Δ* | YNL041C | 116% |
| **1** | ***did4Δ*** | **YKL002W** | **62%** |
| 1 | *gga1Δ* | YDR358W | 81% |
| 1 | *gga2Δ* | YHR108W | 122% |
| **1** | ***glo3Δ*** | **YER122C** | **41%** |
| **1** | ***gos1Δ*** | **YHL031C** | **38%** |
| **1** | ***mon2Δ*** | **YNL297C** | **34%** |
| 1 | *pep5D* | YMR231W | 76% |
| 1 | *rer1Δ* | YCL001W | 151% |
| **1** | ***rgp1Δ*** | **YDR137W** | **56%** |
| **1** | ***ric1Δ*** | **YLR039C** | **47%** |
| **1** | ***sec22Δ*** | **YLR268W** | **49%** |
| **1** | ***stp22Δ*** | **YCL008C** | **13%** |
| **1** | ***vam3Δ*** | **YOR106W** | **75%** |
| **1** | ***vam7Δ*** | **YGL212W** | **39%** |
| **1** | ***vps4Δ*** | **YPR173C** | **55%** |
| **1** | ***vps8Δ*** | **YAL002W** | **58%** |
| **1** | ***vps9Δ*** | **YML097C** | **61%** |
| **1** | ***vps20Δ*** | **YMR077C** | **75%** |
| **1** | ***vps21Δ*** | **YOR089C** | **56%** |
| **1** | ***vps24Δ*** | **YKL041W** | **23%** |
| 1 | *vps27Δ* | YNR006W | 90% |
| **1** | ***vps30Δ*** | **YPL120W** | **47%** |
| **1** | ***vps36Δ*** | **YLR417W** | **29%** |
| **1** | ***vps41Δ*** | **YDR080W** | **52%** |
| **1** | ***ypt6Δ*** | **YLR262C** | **54%** |

| | | | |
|---|---|---|---|
| **2** | ***arf1Δ*** | **YDL192W** | **58%** |
| **2** | ***mon1Δ*** | **YGL124C** | **49%** |
| **2** | ***pep7Δ*** | **YDR323C** | **38%** |
| **2** | ***pep12Δ*** | **YOR036W** | **48%** |
| **2** | ***per1Δ*** | **YCR044C** | **52%** |
| **2** | ***swa2Δ*** | **YDR320C** | **0%** |
| **2** | ***sys1Δ*** | **YJL004C** | **66%** |
| **2** | ***vps1Δ*** | **YKR001C** | **24%** |
| **2** | ***vps3Δ*** | **YDR495C** | **33%** |
| **2** | ***vps29Δ*** | **YHR012W** | **47%** |
| 2 | *vps52Δ* | YDR484W | 109% |
| **2** | ***vps75Δ*** | **YNL246W** | **21%** |
| | | | |
| **3** | ***apl2Δ*** | **YKL135C** | **64%** |
| 3 | *get1D* | YGL020C | n.d. |
| 3 | *get2D* | YER083C | n.d. |
| **3** | ***nhx1Δ*** | **YDR456W** | **20%** |
| **3** | ***pep3Δ*** | **YLR148W** | **46%** |
| 3 | *rvs161D* | YCR009C | n.d. |
| 3 | *rvs167D* | YDR388W | n.d. |
| **3** | ***snx3Δ*** | **YOR357C** | **10%** |
| 3 | *tlg2Δ* | YOL018C | <u>276%</u> |
| **3** | ***vps16Δ*** | **YPL045W** | **45%** |
| **3** | ***vps33Δ*** | **YLR396C** | **47%** |
| 3 | *vps51Δ* | YKR020W | <u>372%</u> |
| 3 | *vps53Δ* | YJL029C | <u>142%</u> |
| 3 | *vps54Δ* | YDR027C | 106% |

Cells were incubated with $^{86}$RbCl as described in the legend to Figure 2. Aliquots were removed at time 0 and at 30 min. Uptake was determined in triplicate. Each strain was tested at least twice on different days; the average of the independent determinations relative to wild type is shown. Results between experiments generally varied by less than 10%. The different mutants were sorted into 3 separate bins: strains exhibiting <75% of wild type uptake were conserved to be defective for uptake (in **bold**). Strains exhibiting >125% of wild type were considered to have excess uptake (<u>underlined</u>) and were examined for $^{86}Rb^{+}$ efflux as described in the text. Strains exhibiting between >75% but <125% of wild type were considered to be indistinguishable from wild type.
