# Supplementary material for: Identification of Yeast Genes Involved in K+ Homeostasis: Loss of Membrane Traffic Genes Affects K+ Uptake
Source: G3 (Bethesda). 2011 Jun 1;1(1):43–56. doi: 10.1534/g3.111.000166 (PMC3276120; doi:10.1534/g3.111.000166)
Supplement: Supporting Information [file supp_1.1.43_TableS4.pdf]

Table S4 Effect of K^+ on CPY Secretion in Membrane Traffic Mutants

Class	Gene	ORF	CPY	CPY on KCl	CPY on Sorb
-	WT	-	-	-	-
1	*arl1Δ*	YBR164C	+++	+/-	+/-
1	*bro1Δ*	YPL084W	++++	-	-
1	*chs5D*	YLR330W		*non-secretor*	
1	*cog5Δ*	YNL051W	++++	+	-
1	*cog6Δ*	YNL041C	++++	+	-
**1**	***did4Δ***	**YKL002W**	**+++**	**-**	**++**
1	*gef1D*	YJR040W	++	-	-
1	*gga1Δ*	YDR358W	+++	-	-
1	*gga2Δ*	YHR108W	+++	-	-
1	*glo3D*	YER122C	+/-	-	-
1	*gos1Δ*	YHL031C	++++	++	+
1	*mdm20D*	YOL076W	+/-	-	-
1	*mon2D*	YNL297C	+/-	+/-	+/-
1	*pep5Δ*	YMR231W	++++	++++	++++
1	*rer1D*	YCL001W		*non-secretor*	
1	*rgp1Δ*	YDR137W	++++	+	+
1	*ric1Δ*	YLR039C	++++	+++	+++
1	*sec22Δ*	YLR268W	+++	+	+
1	*stp22Δ*	YCL008C	++++	-	-
1	*vam3D*	YOR106W	+/-	-	-
1	*vam7Δ*	YGL212W	++++	+++	+++
1	*van1D*	YML115C	++	-	-
1	*vph2Δ*	YKL119C	+++	-	-
**1**	***vps4Δ***	**YPR173C**	**++++**	**+/-**	**+++**
**1**	***vps8Δ***	**YAL002W**	**++++**	**+/-**	**+++**
**1**	***vps9Δ***	**YML097C**	**++++**	**+/-**	**+++**
1	*vps20Δ*	YMR077C	++++	+/-	+/-
1	*vps21Δ*	YOR089C	++++	+	+
**1**	***vps24Δ***	**YKL041W**	**++++**	**+/-**	**+++**
**1**	***vps27Δ***	**YNR006W**	**++++**	**+/-**	**+++**
1	*vps30Δ*	YPL120W	++++	++++	++++
1	*vps36Δ*	YLR417W	++++	+	+
1	*vps41Δ*	YDR080W	++++	++	++
1	*ypt6Δ*	YLR262C	++++	+++	++++

2	*arf1Δ*	YDL192W	++++	+++	+++
2	*mon1D*	YGL124C	+++	+++	++
**2**	***pep7Δ***	**YDR323C**	**++++**	**-**	**++**
**2**	***pep12Δ***	**YOR036W**	**++++**	**-**	**++**
2	*per1Δ*	YCR044C	+++	-	-
2	*swa2Δ*	YDR320C	+++	+	+
2	*sys1Δ*	YJL004C	++++	+	+
**2**	***vps1Δ***	**YKR001C**	**++++**	**+**	**+++**
**2**	***vps3Δ***	**YDR495C**	**++++**	**-**	**+++**
2	*vps29Δ*	YHR012W	++++	++++	++++
2	*vps52Δ*	YDR484W	++++	+++	++++
2	*vps75Δ*	YNL246W	++++	+/-	+/-
3	*nhx1Δ*	YDR456W	++++	++++	++++
3	*pep3Δ*	YLR148W	++++	+++	+++
3	*ptc1D*	YDL006W	++	-	-
3	*snx3D*	YOR357C	++	-	-
3	*tlg2Δ*	YOL018C	++++	++++	++++
3	*vps16Δ*	YPL045W	++++	++++	+++
3	*vps33Δ*	YLR396C	++++	++	++
3	*vps51Δ*	YKR020W	++++	+++	+++
3	*vps53Δ*	YJL029C	++++	++++	++++
3	*vps54Δ*	YDR027C	++++	+++	+++

The membrane traffic mutants in Classes I, II, and III were compared to the strains known to secrete CPY (Bonangelino *et al.*, 2002). We included in our analysis 5 strains obtained in our screen which secrete CPY but do not have GO terms that connote membrane traffic (*gef1D*, *mdm20D*, *van1D*, *vph2D* and *ptc1D;* see Tables S2A). All were grown overnight in rich medium, diluted to 1.0 OD_{600}/ml, then subjected to serial 10-fold dilutions. Cells were spotted onto rich medium without or with the additions of 0.5 M KCl or 1 M sorbitol using a replicator tool and grown overnight at 30°C. The next day, cells were overlaid with a nitrocellulose filter. After 15 -18 h, the filter was removed, washed free of cells, and prepared for Western analysis using a monoclonal anti-CPY antibody (Roberts *et al.*, 1991). Strains are listed by class as in Tables S1A-C and strains in which KCl specifically suppressed secretion are highlighted in bold. - = no secretion, +/- = minimal secretion, + or ++ = moderate secretion, and +++ or ++++ = large amounts of secreted CPY.
